# Andrographolide Analogue Induces Apoptosis and Autophagy Mediated Cell Death in U937 Cells by Inhibition of PI3K/Akt/mTOR Pathway

**DOI:** 10.1371/journal.pone.0139657

**Published:** 2015-10-05

**Authors:** Deepak Kumar, Bimolendu Das, Rupashree Sen, Priyanka Kundu, Alak Manna, Avijit Sarkar, Chinmay Chowdhury, Mitali Chatterjee, Padma Das

**Affiliations:** 1 Cancer Biology and Inflammatory Disorder Division, CSIR-Indian Institute of Chemical Biology, 4, Raja S.C. Mullick Road, Kolkata 700 032, India; 2 Chemistry Division, CSIR-Indian Institute of Chemical Biology, 4, Raja S.C. Mullick Road, Jadavpur, Kolkata 700 032, India; 3 Department of Pharmacology, Institute of Post Graduate Medical Education and Research, 244B, A.J.C. Bose Road, Kolkata 700 020, India; Complutense University, SPAIN

## Abstract

**Background:**

Current chemotherapeutic agents based on apoptosis induction are lacking in desired efficacy. Therefore, there is continuous effort to bring about new dimension in control and gradual eradication of cancer by means of ever evolving therapeutic strategies. Various forms of PCD are being increasingly implicated in anti-cancer therapy and the complex interplay among them is vital for the ultimate fate of proliferating cells. We elaborated and illustrated the underlying mechanism of the most potent Andrographolide analogue (AG–4) mediated action that involved the induction of dual modes of cell death—apoptosis and autophagy in human leukemic U937 cells.

**Principal Findings:**

AG–4 induced cytotoxicity was associated with redox imbalance and apoptosis which involved mitochondrial depolarisation, altered apoptotic protein expressions, activation of the caspase cascade leading to cell cycle arrest. Incubation with caspase inhibitor Z-VAD-fmk or Bax siRNA decreased cytotoxic efficacy of AG–4 emphasising critical roles of caspase and Bax. In addition, AG–4 induced autophagy as evident from LC3-II accumulation, increased Atg protein expressions and autophagosome formation. Pre-treatment with 3-MA or Atg 5 siRNA suppressed the cytotoxic effect of AG–4 implying the pro-death role of autophagy. Furthermore, incubation with Z-VAD-fmk or Bax siRNA subdued AG–4 induced autophagy and pre-treatment with 3-MA or Atg 5 siRNA curbed AG–4 induced apoptosis—implying that apoptosis and autophagy acted as partners in the context of AG–4 mediated action. AG–4 also inhibited PI3K/Akt/mTOR pathway. Inhibition of mTOR or Akt augmented AG–4 induced apoptosis and autophagy signifying its crucial role in its mechanism of action.

**Conclusions:**

Thus, these findings prove the dual ability of AG–4 to induce apoptosis and autophagy which provide a new perspective to it as a potential molecule targeting PCD for future cancer therapeutics.

## Introduction

Leukemia, cancer of blood and bone marrow is one of the most common hemato-oncological disorders caused by the aberrant proliferation of bone marrow derived cells that intrude the bloodstream, lymphoid system precipitating loss of normal bone marrow function and invading distant organs [1]. In recent years, tremendous progress has been made in our understanding of prognosis of leukemia at the cellular and molecular levels. Existing therapeutic protocols have improved patient survival rate. However, alarmingly high numbers of cases still relapse and are inundated by long-term side effects of therapy [2]. Thus, there is an urgent need for novel therapies and chemotherapeutic drugs that target specific signalling pathways which shall eliminate inappropriate cell growth and offer promise of greater specificity coupled with reduced systemic toxicity.

PCD, known to be a crucial process that has an influential role in development, differentiation, cellular homeostasis, elimination of undesired and malignant cells, can be classified according to the morphology of dying cell [3]. Apoptosis, a type I PCD is featured by phosphatidylserine externalization, caspase cascade activation leading to DNA fragmentation [4]. More recently, autophagy, a process conventionally considered as a survival mechanism, has been implicated as type II PCD, and involves engulfment of cytosolic components in *de novo*-synthesized, double membrane enclosed vesicles and subsequent delivery of the enclosed components to lysosomes for degradation [5]. For quite a long time, apoptosis has been considered to be the most important mechanism underlying anti-tumor activity. However, in recent studies it has been revealed that apoptosis is not the sole determinant of destiny of cells [3]. The association between apoptosis and autophagy is multi-faceted and depends on cellular context and the levels of stress involved [6]. They are very closely connected and may cooperate, coexist, or antagonize each other on progressive occurrence of cell death triggered by chemotherapeutic agents. Thus, when cancer cells are subjected to chemotherapeutic treatment, the interplay between the two modes of PCD has tremendous effect on cells and is an essential factor in the determination of the overall fate of cells [7].

Another crucial strategy involved in the process of death induction may be to target aberrantly hyperactivated signaling pathways involved in cellular functions (such as proliferation, survival), and drug resistance of leukemic cells. One such pathway cited in the context of PCD is the PI3K/Akt/mTOR signaling network [8,9]. A major activator of Akt is phosphatidylinositol–3, 4, 5-triphosphate which is generated by PI3K [10]. mTOR is a 289 kDa serine/threonine kinase belonging to PI3K family with two biochemically and functionally diverse complexes, namely mTORC1 and mTORC2 [11]. The inter-relationship among PI3K, Akt and mTOR are regulated by positive and negative feedback loops that their simultaneous hyperactivation gets deterred. However, in most malignancies this pathway is constitutively active leading to inhibition of PCD and promotion of cell survival [12]. Therefore, suppression of PI3K/Akt/mTOR pathway could be of immense potential in the induction of PCD of malignant cells.

Nature is bountiful having provided us with a plethora of natural compounds with therapeutic efficacy. They have attracted worldwide attention since they are contemplated to be safe and pose minimal risk to normal cells [13]. *Andrographis paniculata* (Acanthaceae) is commonly used for the alleviation of a wide spectrum of ailments, which include meningitis, acute hepatitis and other acute inflammatory conditions and is very common for its ethnic usage in India and other Southeast Asian countries. Andrographolide, a diterpenoid lactone isolated from *A*.*paniculata*, has a broad range of pharmacological effects, such as anti-oxidant, anti-inflammatory, anti-HIV, immunomodulatory, hepatoprotective and anti-cancer activities [14–17]. Recent studies have shown that Andrographolide-induced cytotoxicity is attributable to autophagy but not apoptosis in human liver cancer cells [18]. In another study, it has been shown that Andrographolide suppresses autophagy, and sensitizes human cancer cells in a p53-independent manner to cisplatin induced apoptosis [19]. Even though it is a remarkable bio-active molecule, Andrographolide is poorly water soluble which renders it difficult to prepare clinical formulations. In order to develop pharmacophores showing better anti-proliferative efficacy than Andrographolide, we had selected Andrographolide for chemo-selective functionalizations at C14 hydroxyl group. As per our earlier study, we had been able to explain that Andrographolide transformed to an ester derivative (AG–4, previously abbreviated as **6a**, S1 Fig) at C14 hydroxyl substantially improved its solubility and anti-proliferative efficacy [20]. Therefore, we intended to ascertain the molecular mechanism of anti-leukemic effect by AG–4 in-depth, particularly in the induction of various forms of PCD.

Besides inducing apoptosis, in this study it was observed that AG–4 also induced autophagy in U937 human leukemic cells. We, therefore, examined the possible mechanisms underlying apoptosis and autophagy. We further evaluated the effects of AG–4 on the complex interplay between these two types of PCD in U937 cells. Moreover, AG–4 was seen to be able to inhibit PI3K/Akt/mTOR signalling network, one of the most crucial pathways which has profound effect on cellular survival. Thus, this is the first report on the multiple modes of cell death triggered by Andrographolide analogue and its underlying mechanism.

## Materials and Methods

### Ethics statement

Blood samples were obtained following informed written consent from normal healthy volunteers. Ethical approvals for the study and consent procedure were obtained from Internal Review Board (*Ethical Committee on Human Subjects*) of CSIR-Indian Institute of Chemical Biology. All clinical investigations have been conducted according to the principles expressed in the Declaration of Helsinki.

### Materials

All chemicals were obtained from Sigma-Aldrich (St. Louis, Missouri, USA) except Phenazinemethosulphate (PMS, 5915) was purchased from Sisco Research Laboratories (Mumbai, India); 3-(4,5-dimethylthiazol-2-yl)-5-(3-carboxymethoxyphenyl)-2-(4-sulphophenyl)-2H-tetrazolium (MTS), inner salt (Promega, Madison, Wisconsin, USA; G1112). Pen strep (15140), RPMI 1640 (31800022), Heat inactivated fetal bovine serum (FBS, 10082–147), 5,5’,6,6’-tetrachloro–1,1’,3,3’-tetraethylbenzimidazolylcarbocyanine iodide (JC–1, T3168), fluo- 4-acetoxymethyl ester (Fluo-4-AM, 14201), 5-chloromethylfluorescein diacetate (CMFDA, C7025), 5-(and–6)-chloromethyl–2',7'-dichlorodihydrofluorescein diacetate (CM-H_2_DCFDA, C6827) and Lipofectamine 2000 (11668–019) were obtained from Invitrogen (Carlsbad, CA, USA). Caspase–3 (K106-100), Caspase–8 (K113-100), Caspase–9 (K119-100) colorimetric assay kits, mitochondria/cytosol fractionation kit (K256-100), cytochrome c (K257-100) and Annexin V (1001–200) were procured from Biovision (Milpitas, CA, USA). Z-Val-Ala-DL-Asp (methoxy)-fluoromethylketone (Z-VAD-fmk, 550377) was obtained from BD Biosciences (San Jose, CA, USA). Cell Death Detection kit (11684817910) was obtained from Roche (Penzberg, Germany). The antibodies against Bcl–2 (7382), Bcl-xl (8392), Bax (23959), Bad (8044), poly(ADP-ribose) polymerase (PARP, 704470), β-Actin (47778), VDAC 1 (sc–8828) Alkaline phosphatase/Horseradish peroxidase conjugated secondary antibodies and enhanced chemiluminescence kit (2048) were purchased from Santa Cruz Biotechnology (Santa Cruz, CA, USA). The antibodies against Beclin 1 (3495), LC3 (3868), Atg 3 (3415), Atg 5 (8540), Atg 7 (8558), Atg 12 (4180), mTOR (2983), p-mTOR^Ser2481^ (2974), Raptor (2280), Rictor (2114), GβL (3274), Akt (4691), p-Akt^Ser473^ (4060), p-c-Raf^Ser259^ (9421), p-GSK–3β^Ser9^ (5558), p-PDK1^Ser241^ (3438), PI3K(p85) (4257), p-PI3K^p85(Tyr458)/p55(Tyr199)^ (4228), LY294002 (9901) and siRNA against Atg 5 (6345), Bax (6321), Akt (6211), mTOR (6381) and siControl (6568) were procured from Cell Signaling Technology (Inc. Beverly, MA, USA). Cyto-ID^®^ Autophagy detection kit (ENZ-51031-0050) was procured from Enzo life sciences (Farmingdale, NY, USA). R2D 1 strand cDNA synthesis kit (G4641) and 2x qPCR mastermix, SYBR (GCR-5A) were procured from GCC Biotech (Kolkata, India).

### Cell culture

U937 (leukemic monocytic lymphoma), Raji (Burkitt’s lymphoma), MCF–7 (breast adenocarcinoma), HCT–15 (colon carcinoma) were obtained from National Centre for Cell Sciences (NCCS), Pune, India and they were cultured in RPMI 1640 medium (pH = 6.8). Media were supplemented with 10% FBS and antibiotics containing 50 IU/ml penicillin G and 50 μg/ml streptomycin. The cells were incubated at 37°C in a humidified atmosphere containing 5% CO_2_ and subcultured every 72 h using an inoculum of 5x10^5^ cells/ml.

### Isolation of human peripheral blood mononuclear cells

PBMC were isolated from anticoagulated blood by density gradient centrifugation on an equal volume of Ficoll-Hypaque (Histopaque–1077) at 400 x g for 30 mins. PBMC were harvested from the interface, washed twice in PBS (0.01 M, pH 7.4) and resuspended in RPMI–1640 medium supplemented with penicillin G (50 IU/ml), streptomycin (50 μg/ml) and 10% FBS (Al Omar et al., 2012). Cell viability was confirmed by trypan blue exclusion (>95%).

### Isolation and characterization of Andrographolide derivative

Andrographolide **1** (AG–1) was isolated with good yield from the leaves of *Andrographis paniculata* and used as starting material for analogue synthesis. The C3 and C19 hydroxyl groups were protected as 3,19-isopropylidene-andrographolide **2** which underwent chemo-selective succinylation at C 14 hydroxy to furnish the intermediate compound **3**. Product **3** was converted easily to andrographolide-14-α-*O*-succinate **4** upon treatment with aqueous acetic acid (3:7). The targeted product **4** (AG–4) was purified through usual silica gel (100–200 mesh) chromatography (ethyl acetate-petroleum ether) followed by high pressure liquid chromatrography (S1 Fig) [20].

### Cell viability assay

Cell viability was evaluated in U937, Raji, HCT–15 and MCF–7 cells and PBMC using MTS-PMS assay as described before [20]. Briefly, log phase cells (2.5–5.0x10^4^/200 μL of RPMI 1640 medium/well) were seeded in 96-well tissue culture plates and incubated with AG–4 (0–50 μM) for 48 h at 37°C, 5% CO_2_. The time-dependent nature of cytotoxicity of AG–4 in U937 cells was also evaluated by incubating with AG–4 (0–50 μM) for 24, 48, 72 h. Following treatment, MTS (2.0 mg/ml) and PMS (0.92 mg/ml) were added in a ratio of 10:1, incubated for 3 h at 37°C, resultant absorbance was measured at 490 nm in an ELISA Reader (BIO RAD). Accordingly, the specific absorbance that represented formazan production was calculated by subtraction of background absorbance from total absorbance. The mean percentage viability was calculated as follows:

$$\frac{\text{Mean specific absorbance of treated cells }\times \text{100}}{\text{Mean specific absorbance of untreated cells}}$$

The results were expressed as IC_50_ values, i.e. the concentration that inhibited 50% of cell growth, which was enumerated by graphical extrapolation using Graph pad prism software (version 5, Graph Pad Software Inc. San Diego, CA, USA). Each experiment was performed at least three times and in duplicate.

### Apoptosis assay

The percentage of apoptotic cells was ascertained by dual staining of cells with Annexin V and propidium iodide [20]. U937 cells (2.5 x 10^5^/ml) were incubated with or without AG–4 (5.4 μM, 48 h) in presence or absence of various inhibitors at 37°C, 5% CO_2_. Cells were then washed twice in PBS and resuspended in Annexin V binding buffer (10 mM HEPES, 140 mM NaCl, 2.5 mM CaCl_2_; pH 7.4). Annexin V-FITC was then added according to the manufacturer’s instructions and incubated for 15 min under dark conditions at 25°C. Propium iodide (0.1 μg/ml) was added just prior to acquisition. Data was acquired using a FACS Aria flow cytometer (Becton Dickinson) at an excitation wavelength of 488 nm and an emission wavelength of 530 nm and analyzed with BD FACS Diva software (Becton Dickinson).

### Determination of intracellular reactive oxygen species

The generation of ROS production in control and AG–4 treated U937 cells was monitored by using the cell permeable fluorescent probe CM-H_2_DCFDA. This dye is hydrolyzed by nonspecific intracellular esterase and oxidized by cellular peroxides to form fluorescent compound DCF [21]. Thus, the fluorescence intensity is proportional to the quantum of ROS generated by the cells. Briefly, U937 cells were exposed to IC_50_ concentration of AG–4 (5.4 μM, 0–3 h), washed with PBS and then loaded with CM-H_2_DCFDA (5 μM) for 30 min at 37°C in PBS. Subsequently, cells were removed, washed and resuspended in PBS and analyzed for DCF fluorescence. To confirm the elevated levels of ROS induced by AG–4, for the inhibition of ROS generation, cells were co-incubated with AG–4 (5.4 μM) and anti-oxidant NAC (2.5 mM) for 3 h. Cells were then similarly processed. Data acquisition was done on a FACS Calibur flow cytometer (Becton Dickinson, USA) at excitation wavelength of 492 nm and emission wavelength of 517 nm. The data was analyzed by BD CellQuest Pro software. To corroborate the non-toxic effect of AG–4 in healthy cells, PBMC (2.5 x 10^5^/ml) were incubated with AG–4 (5.4 μM, 0–3 h) and ROS was similarly quantified. To study the contribution of ROS in AG–4 induced death, U937 cells (5.0x10^4^/200 μL of RPMI 1640 medium/well) were seeded in 96 well tissue culture plates followed by co-incubation with AG–4 (0–50 μM) and NAC (2.5 mM) for 48 h at 37°C. Subsequently, IC_50_ values were evaluated by the MTS-PMS assay. In additional set of experiment, in order to assess the role of ROS in AG–4 induced apoptosis, cells were co-incubated with AG–4 (5.4 μM) and NAC (2.5 mm) for 48 h followed by apoptosis measurement by Annexin V assay.

### Measurement of non-protein thiols

Non-protein thiols were measured as previously described [22]. Briefly, log phase U937 cells were incubated with AG–4 (5.4 μM, 0–3 h). The cells were then washed with PBS, incubated with CMFDA for 15 min at 37°C in the dark and fluorescence acquired on a FACS Calibur using forward vs side scatter to gate the cell population and a FL1 histogram to quantify fluorescence of viable cells. The subsequent analyses were done using CellQuest Pro software.

### Measurement of mitochondrial transmembrane potential

The change in MMP was monitored by using JC–1, a cell permeable, cationic and lipophilic dye that freely permeates the mitochondrial membrane and forms J aggregates that fluoresce red; accordingly, viable cells stained with JC–1 exhibit a pronounced red fluorescence. Following an apoptotic stimulus, the resultant decrease in the mitochondrial membrane potential prevents JC–1 from entering the mitochondria and remains as monomers in the cytosol that emits a predominantly green fluorescence. Therefore, the ratio of J-aggregates/monomers serves as an effective indicator of the cellular MMP. Briefly, U937 cells were incubated with AG–4 (5.4 μM) for 12, 24 and 48 h at 37°C, 5% CO_2_. The cells were then washed with PBS, incubated with JC–1 for 15 min at 20–25°C in the dark. Cells were acquired on a FACS Calibur on the basis of quadrant dot plot to determine monomers and J aggregates and analysed using Cell Quest Pro software. To set quadrants, U937 cells were treated with H_2_O_2_ (15 mM; 37°C; 15 min), representing cells with depolarized mitochondrial membrane potential.

### Intracellular Ca^2+^ measurement

Intracellular Ca^2+^ was measured using the fluorescent probe Fluo–4 AM as previously described [23]. Briefly, the cells were preloaded with Fluo–4 AM (cell membrane permeable fluorescent dye) in loading media (2.5 μM Fluo–4 AM; 0.02% pluronic acid F–127, 0.25 mM sulphinpyrazone in RPMI 1640 medium) for 30 min at 37°C. The cells were then washed with medium containing 0.25 mM sulphinpyrazone and re-suspended in the same media (0.25 mM sulphinpyrazone). Subsequently, cells were incubated with AG–4 (5.4 μM) and rapid kinetic measurement of fluorescence was performed by flow cytometry. In parallel, cells were incubated with a Ca^2+^ ionophore (Ionomycin; 1 μM) and specificity confirmed by addition of a chelating agent EGTA (3 mM).

### Preparation of cytosolic and mitochondrial extracts

Isolation of highly enriched mitochondrial and cytosolic fraction of cells was performed using a mitochondria/cytosol fractionation kit. Briefly, U937 cells were treated with AG–4 (5.4 μM), washed twice in ice-cold PBS, resuspended in cytosol extraction buffer containing DTT and protease inhibitors and incubated on ice for 10 min. The cells were homogenized on ice, centrifuged (700 x g for 10 min at 4°C) and the resultant supernatant was then centrifuged at 10,000 x g for 30 min at 4°C. The supernatant was collected as the cytosolic fraction and the pellet containing the mitochondria was resuspended in mitochondria extraction buffer containing DTT and protease inhibitors and stored at -80°C until further use.

### Western blot analysis

Total cellular proteins were isolated from AG–4 treated and untreated cells (5x10^6^) using lysis buffer (50 mM Tris–HCl pH 7.4, 150 mM NaCl, 1 mM ethylenediaminetetraacetic acid, 1 mM EGTA, 1μg/ml protease inhibitor cocktail, 5 mM phenylmethylsulfonyl fluoride and 1 mM dithiothreitol containing 1% Triton X–100). Cells were lysed, centrifuged for 10 min at 4°C at 10,000 x g and protein concentration was estimated [24]. Electrophoretic separations (50 μg/lane) were carried out on 10% SDS-polyacrylamide gel electrophoresis, and electrotransferred onto a polyvinylidene fluoride (PVDF) membrane. Blots were blocked for 1 h at 37°C in 20 mM Tris-HCl, pH 7.4, 150 mM NaCl, 0.02% Tween 20 (TBST) containing 5% skimmed milk and probed using 1:1000 dilution of appropriate primary antibodies (Bad, Bax, Bcl–2, Bcl-xl, PARP, cytochrome c, Beclin–1, VDAC 1, Atg 3, Atg 5, Atg 7, Atg 12, mTOR, p-mTOR^Ser2481^, Raptor, Rictor, GβL, Akt, p-Akt^Ser473^, p-c-Raf^Ser259^, p-GSK–3β^Ser9^, PI3K(p85), p-PI3K^p85(Tyr458)/p55(Tyr199)^, p-PDK1^Ser241^, β-Actin) by incubating overnight at 4°C. The membranes were washed thrice with TBST, incubated with alkaline phosphatase/Horseradish peroxidase conjugated secondary antibody and the bands visualized using a 5-bromo-4-chloro-3-indolyl phosphate/nitro blue tetrazolium substrate or enhanced chemiluminescence kit. Equal loading of samples was confirmed using β-Actin as a control.

### Measurement of caspase activity

The enzymatic activity of caspase -3, -8, -9 induced by AG–4 was assayed using colorimetric assay kits as per the manufacturer’s instructions. Briefly, cell lysates were prepared after their respective treatment with AG–4. Assays were performed in 96-well microtiter plates by incubating cell lysates (100 μg protein in 50 μl lysis buffer) in 50 μl of reaction buffer (provided by the manufacturer containing 10 mM DTT) and incubated with specific colorimetric peptide substrates (N-acetyl-Asp-Glu-Val-Asp-p-nitroanilide (Ac-DEVD-pNA) for Caspase–3, N-acetyl-Ile-Glu-Thr-Asp-p-nitroanilide (Ac-IETD-pNA) for Caspase–8, N-acetyl-Leu-Glu-His-Asp-p-nitroanilide (Ac-LEHD-pNA) for Caspase–9; 4 mM, 5 μl) at 37°C for 5 h. The emission of pNA was measured at 405 nm in an ELISA reader. Results were expressed as change in fold of activity compared to control.

To investigate into the contribution of caspases towards AG–4 induced cytotoxicity, U937 cells were seeded with AG–4 (0–50 μM) and Z-VAD-fmk (20 μM), a pan caspase inhibitor. All sets were incubated at 37°C for 48 h. Subsequently, IC_50_ values were determined by MTS-PMS assay. In another set of experiment, in order to assess the role of caspase activation in AG–4 induced apoptosis, cells were co-incubated with AG–4 (5.4 μM) and Z-VAD-fmk (20 μM) for 48 h followed by apoptosis measurement by Annexin V assay.

### Terminal DeoxyribonucleotidylTransferase-Mediated dUTP Nick-End Labeling assay

The extent of DNA fragmentation was estimated using the TUNEL assay. U937 cells (2.5 x 10^5^/ml) were treated with AG–4 (5.4 μM) for 48 h at 37°C, 5% CO_2_ and cellular DNA was stained according to the manufacturer’s instructions. Briefly, cells were fixed in paraformaldehyde (2% in PBS) and kept on ice for 1 h, centrifuged (2000 x g, 5 min), resuspended in PBS (10 μl) spotted and air dried; they were washed with PBS and incubated with H_2_O_2_ (3% in MeOH) for 10 min at 4°C. The slides were washed with PBS, placed on ice and permeabilized with freshly prepared, chilled Na-Citrate (0.1%) in Triton X–100 (0.1%) solution for 2 min. Cells were washed twice with PBS, reacted with enzyme terminal deoxy transferase (TdT) and nucleotide mixture was added. The slides were then incubated in a humidified chamber at 37°C for 1 h, washed with PBS and convertor POD, (antifluorescein antibody conjugated with horseradish peroxidase, 25 μl) added and incubated for 30 min at 37°C. Finally, the substrate Diaminobenzidine (25 μl) was added, slides were kept at 4°C for 10 min, washed with deionised water and observed microscopically under oil immersion objective; at least 20 microscopic fields were observed for each sample (Olympus, Singapore).

### Flow Cytometric determination of cell cycle arrest

The status of cell cycle was determined by flow cytometry with DNA staining to reveal the total amount of DNA. Approximately, 1x10^6^ cells were treated with an IC_50_ dose of AG–4 (5.4 μM) for 12, 24 and 48 h, thereafter cells were fixed in chilled ethanol (70%) and kept at -20°C until analysis [25]. The cells were then washed with PBS, digested with DNase free RNase (200 μg/ml) for 1 h at 37°C. Cells were stained with propidium iodide (80 μg/ml), kept for 20 min at 20–25°C in the dark. The percentage of cells in each phase of the cell cycle was determined by a FACS Calibur and analyzed using CellQuest Pro software. The fluorescence intensity of sub G_0_/G_1_ cell fraction represented the apoptotic cell population.

### Alteration of LC3

During autophagy, the cytoplasmic form of microtubule-associated protein light chain 3 (LC3-I) is processed to its membrane associated form LC3-II and recruited to the autophagosomes. Therefore, LC3-II serves as a widely used marker for autophagosomes [26]. Briefly, whole cell lysates were prepared from control and AG–4 treated (5.4 μM, 0–48 h) [with or without co-treatment with, 3-MA (10 mM, 4 h), Bafilomycin A1 (50 nM), E64d (10 μg/ml) with pepstatin A (10 μg/ml), Chloroquine (5 μM)] U937 cells, protein concentration estimated and western blotting analysis was done as described above.

### Quantitative Real-time PCR

Total RNA was isolated with Trizol reagent and 1 μg of the total RNA was reverse-transcribed into cDNA with M-MLV reverse transcriptase in the presence of oligo (dT)12-18. Real-time PCR was performed in triplicate with SYBR Green master mix for 10 min at 95°C for initial denaturation, followed by 40 cycles of segments of 95°C for 15 sec, 58°C for 30 sec and 72°C for 30 sec in the StepOne™ Real-Time PCR System (Applied Biosystem). The primer used to amplify the LC3 gene is 5'-ATG CCG TCG GAG AAG ACC TT–3' (forward) and 5'-TTA CAC TGA CAA TTT CAT CCC G–3' (reverse). The expression levels of the LC3 gene was normalized against the expression levels of the housekeeping gene gapdh.

### Determination of autophagy (Cyto-ID staining)

Flow cytometric analysis of Cyto-ID Green Detection Reagent stained cells was performed according to manufacturer’s protocol (Cyto-ID Autophagy Detection Kit, ENZ-51031-K200, Enzo Life Sciences). In brief, U937 cells were washed with PBS, stained with Cyto-ID for 30 min at 37°C, washed with 1X Assay Buffer and resuspended in 1X Assay Buffer. Cell suspension was quantified by flow cytometry.

### Supravital cell staining with AO for detection of AVO

Autophagy is the process of sequestrating cytoplasmic proteins and organelles into the lytic component and characterized by the formation and promotion of AVO. To detect AVO formation in AG–4 treated cells, we performed the vital staining with AO. In cells stained with AO, the cytoplasm and nucleoli emit green fluorescence whereas the acidic compartments emit red fluorescence, whose intensity is proportional to the degree of acidity [27]. Control and AG–4 (5.4 μM, 0–48 h) treated U937 cells were washed in PBS and were stained with AO (1 μg/ml) for a period of 15 min. Cells were resuspended in PBS and observed under a fluorescence microscope (Carl Zeiss, Germany) at an excitation of 488 nm and emission of 530 nm and 650 nm. AVO formation was also quantified by flow cytometry after the cells were stained with AO (1 μg/ml, 15 min) and resuspended in 500 μl of PBS. To confirm that the change in fluorescence is due to autophagy induced by AG–4, U937 cells were pre-incubated with 3-MA (10 mM for 4 h) followed by treatment with AG–4 (5.4 μM, 48 h) and evaluated by fluorescence microscopy or flow cytometry.

### Transmission electron microscopy

Control and AG–4 treated U937 cells were fixed with a solution containing 2.5% glutaraldehyde and 2% paraformaldehyde in 0.1 M phosphate buffer (pH 7.4) for 1 h at 4°C. After fixation, cells were rinsed in PBS, and postfixed in 1% osmium tetroxide in the same buffer for 2 h, dehydrated in graded acetone and embedded in araldite CY212. Semi-thin sections were stained with toluidine blue (0.5%, 5 min), and observed under a light microscope (Olympus). Ultrathin sections were stained with 2% uranyl acetate and Reynold’s lead citrate, and observed with a transmission electron microscope (Technai G2) [28].

### Small interfering RNA (siRNA) and transient transfection

Cells were seeded in 6 well tissue culture plates and transfected the next day with siAtg 5, siBax, siAkt, simTOR or siControl using Lipofectamine 2000. Transfection was performed according to manufacturer’s instructions. After 48–72 h, cells were treated with AG–4 (5.4 μM) for 48 h for subsequent western blot analysis and viability assay.

### Statistical analysis

The statistically significant differences between control and drug treated cells were calculated using one way ANOVA and two way ANOVA. Multiple comparisons were made between different treatments (analysis of variance) using Graph Pad Prism Software (version 5, GraphPad Software Inc, San Diego, CA, USA) and an error protecting the multiple comparison procedure, namely Tukey’s multiple comparison test. p<0.05 was considered as statistically significant. All data were expressed as mean± SEM/SD.

## Results

### Dose- and time-dependent anti-proliferative efficacy of AG–4

**Fig 1:**
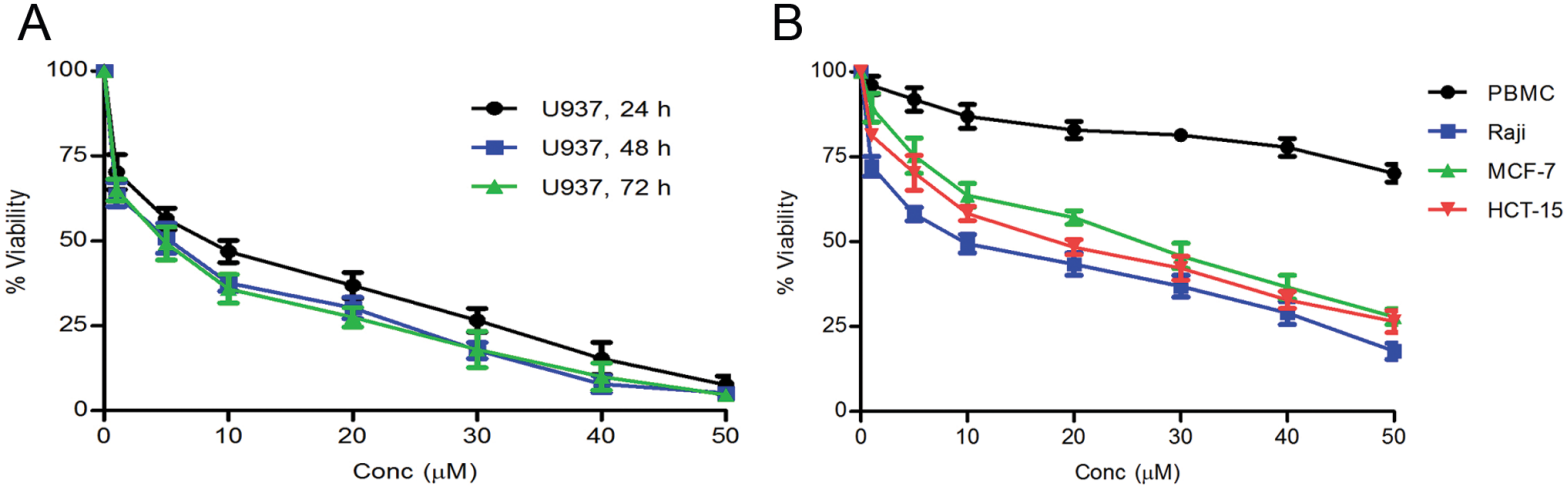
AG–4 induces anti-proliferative effect. (A) Time-dependent effect in U937 cells. U937 cells were treated with AG–4 (0–50 μM) for 24, 48 or 72 hours. Cell death was assessed by MTS-PMS assay. Each data point represents the mean±SEM of at least three independent experiments in duplicate. (B) Effect on other cells. Human cancer cell lines (Raji, MCF–7, HCT–15) and healthy human PBMC were incubated with AG–4 (0–50 μM) for 48 h. Cell death was assessed by MTS-PMS assay. Each data point represents the mean±SEM of at least three independent experiments in duplicate.

Viability of U937 cells was observed by MTS-PMS assay following treatment with different concentrations of AG–4 (0–50 μM) for 24, 48, 72 h. Treatment with AG–4 significantly decreased the proliferation of U937 cells in depending on dose- and time of exposure. After treatment of cells for 24 h, it showed that the inhibitory concentration of AG–4 reached 8.7±1.1 μM (Mean IC_50_±SEM). A prolonged incubation for 48 h and 72 h, the cell growth inhibitory concentration decreased to 5.4±0.7 μM and 4.6±0.9 μM respectively (Fig 1A). Since, there was no significant reduction in IC_50_ values after 48 h, subsequent studies have been conducted using 5.4 μM. Besides, anti-proliferative effect of AG–4 was also investigated in Raji, MCF–7, HCT–15 cells and the IC_50_ was found out to be 10.0, 17.9, 25.5 μM respectively (Fig 1B) implying that AG–4 was selective in action and most potent in U937 cells. We further analyzed the effects of AG–4 on PBMC isolated from healthy individuals. AG–4 was unable to inhibit proliferation of PBMC even at a concentration of 50 μM (Fig 1B), substantiating its non-toxicity towards healthy cells. Biological inertness of DMSO (0.01%) present in the highest concentration used (50 μM) was confirmed as it failed to demonstrate any effect on cell viability.

### AG–4 induces pro-oxidant activity in U937 cells

**Fig 2:**
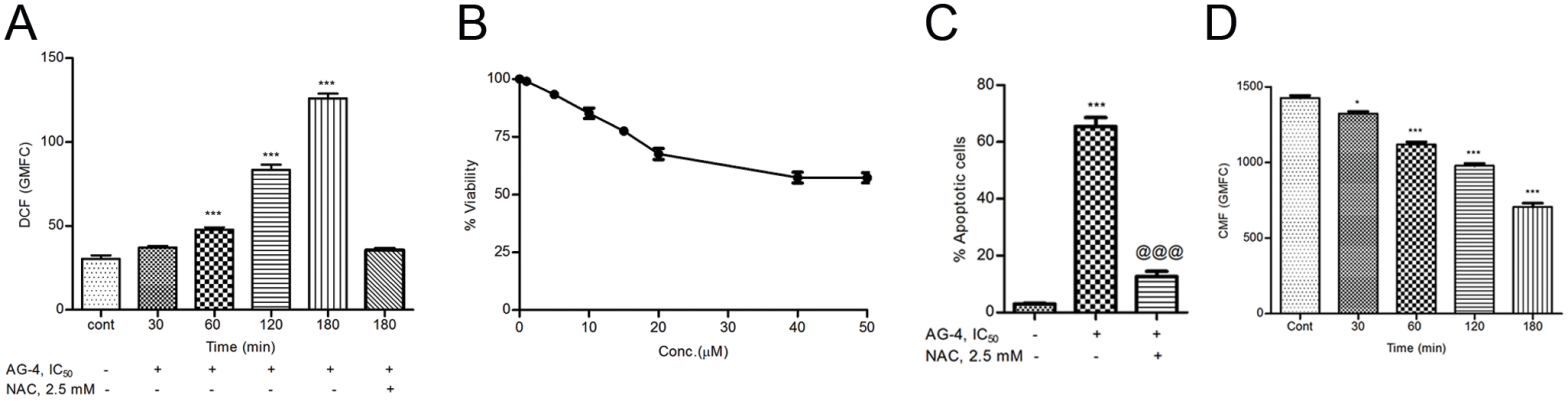
AG–4 induces redox imbalance in U937 cells. (A) Effect of AG–4 on ROS generation. U937 cells were treated with AG–4 (5.4 μM, 0–3 h), stained with CM-H_2_DCFDA and ROS was measured by flow cytometry. For the inhibition of ROS generation, cells were co-treated with NAC (2.5 mM) and AG–4 (5.4 μM, 3 h) and ROS was similarly quantified. Data represent mean GMFC±SEM of three independent experiments (***p<0.001, as compared with control). (B) Effect of antioxidant on survival of U937 cells. Cells were co-incubated with AG–4 (0–50 μM) and NAC (2.5 mM) for 48 h and MTS-PMS assay was performed. Each point corresponds to the mean ± SEM of at least three experiments in duplicate. (C) Effect of antioxidant on AG–4 induced apoptosis. Cells were treated with AG–4 (5.4 μM, 48 h) in presence or absence of NAC (2.5 mM). They were co-stained with Annexin V-FITC and PI followed by analysis for phosphatidylserine externalization using flow cytometry as described in materials and methods. Histograms represent percentage apoptotic cells and have been derived from at least three experiments (***p<0.001, compared to control cells; @@@ p<0.001, compared to only AG–4 treated cells). (D) Effect of AG–4 on level of non-protein thiols. U937 cells treated with AG–4 (5.4 μM, 0–3 h) were labelled with CMFDA and analysed for fluorescence. Data are expressed as mean GMFC±SEM of three independent experiments (*p<0.05, ***p<0.001, as compared with control).

ROS are implicated as initial mediators of PCD that inflicts damage on intracellular components such as DNA, proteins and amino acids [29]. As shown in Fig 2A, a time dependent increase of ROS levels was detected in U937 cells, maximum being at 3 h post AG–4 treatment. The mean±SEM of GMFC representing baseline ROS was 30.56±2.0, 25.48±4.9 in U937 cells and PBMC respectively. This progressively increased to 126.0±2.8 (***p<0.001; about 4-fold increase at 3 h compared to the basal level) in U937 cells. On the other hand, in PBMC, ROS levels increased inappreciably to 37.45±3.98 implying the non-toxicity of AG–4 towards healthy cells. Co-treatment of NAC (2.5 mM), a well known antioxidant and AG–4 treatment (5.4 μM) for 3 h inhibited ROS production and GMFC level decreased to 35.64±0.97 signifying that the increase in fluorescence was due to the ability of AG–4 to produce ROS. Furthermore, co-incubation of cells with NAC and AG–4 (0–50 μM) for 48 h increased IC_50_ value from 5.4 μM to >50 μM (Fig 2B) and percentage of apoptotic cells decreased significantly (Fig 2C). Therefore, ROS generated did contribute towards AG–4 induced demise of U937 leukemic cells.

The said ROS generally followed an imbalance in cellular redox homeostasis in the initial phase of apoptosis and was also seen to have been accompanied by a reduction in cellular thiols. For ascertaining the impact of AG–4 on levels of non-protein thiols, CMFDA was used which reacts with non-protein thiols group to form a non-permeable fluorescent product that is retained within cells. Thus, the fluorescence that was generated is an indicator of cellular non-protein thiol levels. As per our findings, we deduced that AG–4 treatment resulted in reduction of non-protein thiol levels with increase in time, mean±SEM of GMFC in untreated cells was found to be 1425.60±16.33, which with the addition of AG–4 progressively decreased to 705.70±24.61 at 3 h (***p<0.001, Fig 2D). Taken together, these results indicated that AG–4 caused oxidative imbalance in U937 cells which is a key factor triggering the anti-proliferative activity of AG–4.

### AG–4 induced apoptosis involves mitochondria mediated pathway

**Fig 3:**
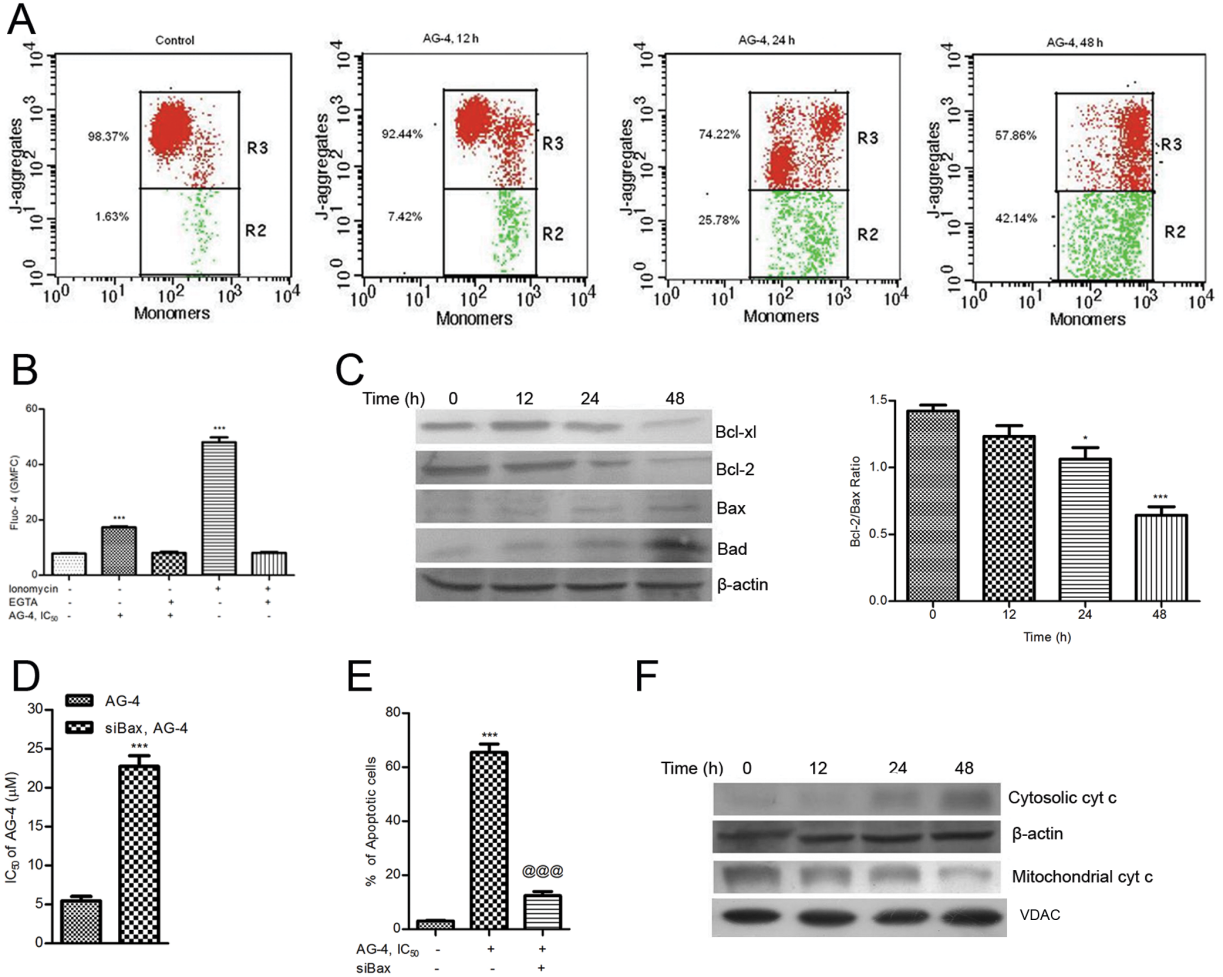
Involvement of mitochondrial pathway in AG–4 induced apoptosis. (A) Loss of mitochondrial membrane potential. Cells were incubated with AG–4 (5.4 μM, 0–48 h) and loaded with JC–1 for flow cytometric analysis of mitochondria transmembrane potential. Data is a representative of three different experiments. (B) Effect on intracellular Ca^2+^. Cells preloaded with Fluo–4 AM were incubated with AG–4 (5.4 μM). The flow cytometric measurement of free cytosolic Ca^2+^ levels was seen as a fluorescent signal. Data are expressed as mean GMFC±SEM of three independent experiments (***p<0.001, as compared with control). (C) Altered expression levels of pro- and anti-apoptotic proteins. Whole cell extracts were made from control and AG–4 (5.4 μM, 0–48 h) treated cells and subjected to western blot analysis for Bcl–2, Bcl-xl, Bax, Bad. Analysis was confirmed with three different sets of extracts. β-actin served as a loading control. Histogram shows the time dependent decrease in Bcl–2/Bax ratio (*p<0.05, ***p<0.001, as compared with control). (D) Contribution of Bax in AG–4 induced cytotoxicity. Cells were transfected with siBax for 48 h followed by treatment with AG–4 (0–50 μM, 48 h). Cell viability was determined by MTS-PMS assay. Results are expressed as IC_50_ (mean ± SEM) from three independent experiments (***p<0.001, compared to only AG–4 treated cells). (E) Contribution of Bax in AG–4 induced apoptosis. Cells were transfected with siBax for 48 h followed by treatment with AG–4 (0–50 μM, 48 h). The percentage of apoptotic cells was determined by Annexin V and propidium iodide dual staining. Results are expressed as mean ± SEM from three independent experiments (***p<0.001, compared to control cells; @@@ p<0.001 compared to only AG–4 treated cells). (F) Effect on cytochrome c release. Cytoplasmic and mitochondrial fractions were prepared from control and AG–4 treated (5.4 μM, 0–48 h) cells using mitochondria/cytosol fractionation kit as described in materials and methods and cytochrome c was analyzed by Western blotting. Data shown are from one of the three experiments.

Sustenance of mitochondrial transmembrane potential is necessary for cellular survival and its loss is regarded as a vital event in cell death induced by therapeutic agents [8]. Apoptosis usually involves disruption of mitochondrial membrane integrity which is critical for cell death [30,31]. Also, high intracellular ROS normally disrupts mitochondrial membrane potential. Therefore, we attempted to investigate and ascertain the effect of AG–4 on mitochondrial membrane potential of cells using JC–1, a cationic lipophilic dye which selectively gets incorporated in mitochondria and can reversibly change colour depending on the membrane potential. This differentiates apoptotic cells from the viable ones. JC–1 fluorescence was quantitatively determined using flow cytometry having estimated percentage (mean±SEM) of gated population in two gates i.e. R2 and R3, wherein R_2_ represented the apoptotic cells (monomers) while R3 comprised of the viable cells (J-aggregates). In viable cells, the percentage of cells present in gates R3 and R2 was found to be 98.37±0.42% and 1.63±0.44%, respectively. With AG–4 (5.4 μM) treatment, there had been a progressively time dependent increase in R2 gated population, for instance it was 42.14±3.38% at 48 h (Fig 3A). This could also be regarded as the ratio of J-aggregates/monomers which in control cells was 60.4. With AG–4 addition, this declined to 1.37 after 48 h implying the occurrence of mitochondrial depolarization by AG–4.

Alterations in intracellular Ca^2+^ in response to loss in MMP has been a well noted phenomenon [32]. For the purpose of determining the elevation of Ca^2+^ release in apoptosis induced by AG–4, U937 cells were stained with fluorescent probe Fluo–4 AM and detected by flow cytometry. On addition of Ionomycin, a known effective Ca^2+^ ionophore, intracellular Ca^2+^ was seen to have increased significantly (***p<0.001) whereas on treating cells with Ionomycin along with EGTA (a chelating agent), the same decreased which confirmed assay specificity in U937 cells. As shown in Fig 3B, control cells maintained a stable and steady concentration of intracellular Ca^2+^ whereas treatment with AG–4 resulted in 2.2 fold enhancement (***p<0.001) in cytosolic calcium levels.

Among the prominent regulators of mitochondrial integrity Bcl–2 family members deserve special mention. Interplay among pro-apoptotic (Bax, Bad) and anti-apoptotic (Bcl–2, Bcl-xl) proteins influences the sensitivity of cancer cells to drug-induced apoptosis [32,33]. We thus examined the effect of AG–4 on key pro-apoptotic and anti-apoptotic proteins by western blotting. As evident from Fig 3C, a time dependent elevation in Bax and Bad expression levels occurred concomitantly with depletion in Bcl–2 and Bcl-xl levels. This was reflected in the gradual reduction in Bcl–2/Bax ratio with time (*p<0.05, ***p<0.001; Fig 3C). Since elevation of Bax levels was observed after treatment with AG–4, it could be hypothesized that silencing of Bax expression shall reduce AG–4 induced cytotoxicity and thereby apoptosis. Bax expression was significantly silenced following 48 h of transfection using siRNA targeted against Bax (S2 Fig). When Bax silenced cells were subjected to AG–4 treatment for 48 h, IC_50_ of AG–4 increased to 22.7 μM (Fig 3D) and the percentage of apoptotic cells demonstrated a sharp decline to 10% (Fig 3E). This clearly emphasized the role of Bax in AG–4 mediated death. A decrease in MMP was found to be responsible for recruitment of the mitochondrial pathway of apoptotic signalling together with the release of cytochrome c [32]. As shown in Fig 3F, treatment with AG–4 for increasing time durations caused a decrease in mitochondrial cytochrome c coupled with a concomitant rise in cytosolic cytochrome c. Altogether; these data indicated that AG–4 induced apoptosis in U937 cells through the mitochondrial pathway.

### Activation of caspases and cleavage of PARP by AG–4

**Fig 4:**
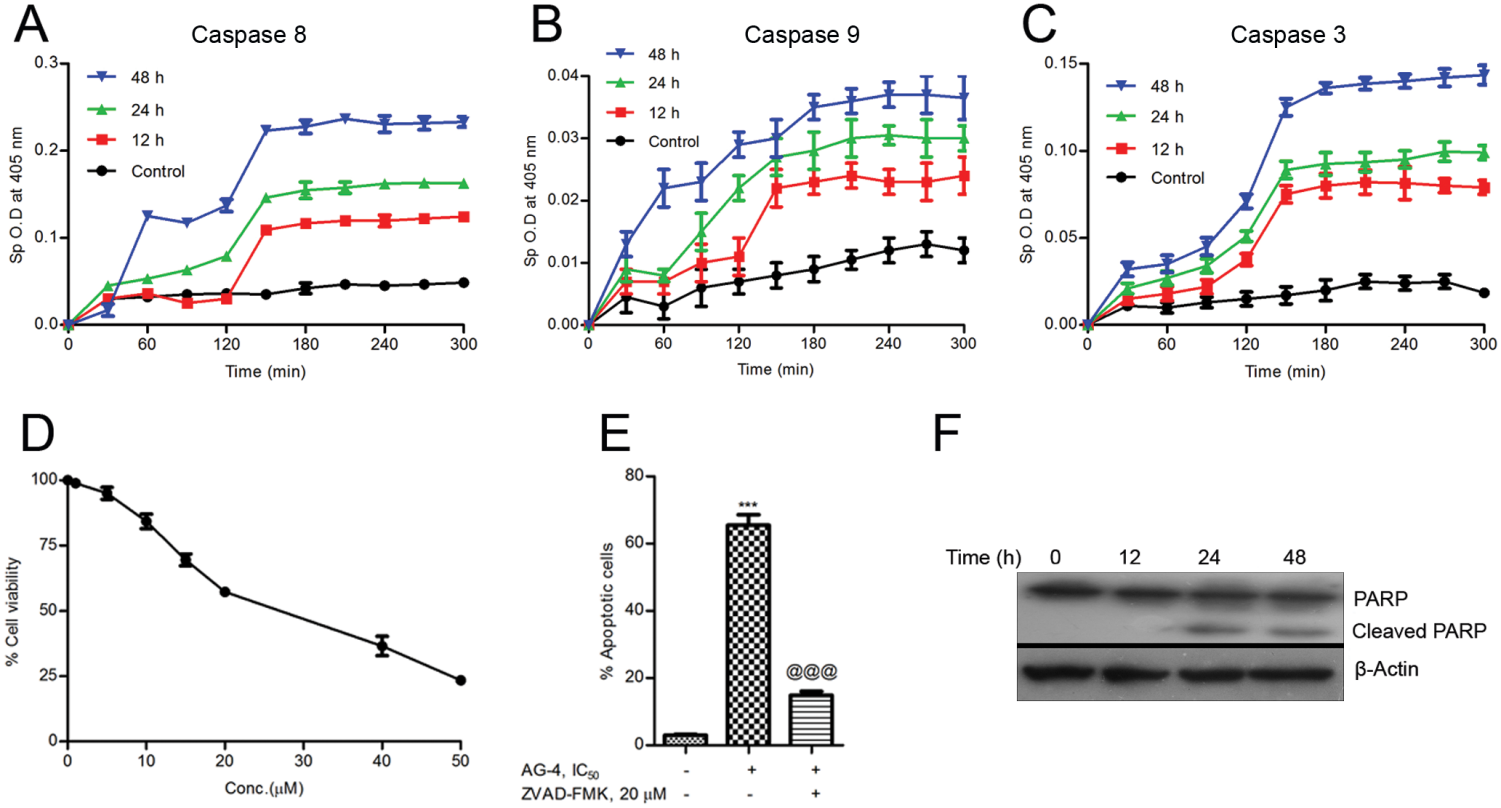
Activation of Caspase -8, -9, -3 by AG–4. Activity of caspase–8 (A), -9 (B), -3 (C) was measured in control and AG–4 treated (5.4 μM, 0–48 h) U937 cells using colorimetric tetrapeptide substrates. Results are expressed as mean±SEM from three independent experiments. (D) Effect of caspase inhibitor on cell viability. U937 cells were incubated with AG–4 (0–50 μM) along with pan caspase inhibitor, Z-VAD-fmk (20 μM) for 48 h and MTS-PMS assay was performed. Each point corresponds to the mean±SEM of at least three experiments in duplicate. (E) Effect of caspase inhibitor on AG–4 induced apoptosis. Cells were treated with AG–4 (0–50 μM, 48 h) with or without Z-VAD-fmk (20 μM). They were co-stained with Annexin V-FITC and PI followed by analysis using flow cytometry. Histograms represent percentage of apoptotic cells and have been derived from at least three experiments (***p<0.001, compared to control cells; @@@ p<0.001, compared to only AG–4 treated cells). (F) AG–4 enhances PARP cleavage. PARP cleavage was evaluated by Western blotting analysis in extracts of control and AG–4 (5.4 μM, 0–48 h) treated cells. Data was confirmed with three different sets of experiments.

It is well known that caspases are evolutionarily conserved executioners of PCD. When mitochondria release cytochrome c following altered mitochondrial permeability it could activate caspase cascade [32]. This promoted us to measure the activity of caspase–3, -8 and -9 in cell lysates of control and AG–4 treated cells by quantitative detection of colorimetric tetrapeptide substrates. The activities of caspase–3, -8 and -9 in treated cells demonstrated a rise with longer durations of treatment; the maximum being after 48 h treatment (Fig 4A–4C). A significant exponential surge in the activity of caspase–8 (~3 times rise as compared to control), caspase–9 (~5 times rise as compared to control) and caspase–3 (~8 times rise as compared to control) was noted up to 3 h and thereafter change in activity remained constant. Substantial activation of caspases during apoptosis was further corroborated by incubation of cells with Z-VAD-fmk believed to be a potent pan caspase inhibitor. It revealed that IC_50_ value increased to 27 μM from 5.4 μM (Fig 4D) validating the crucial role of caspases. Furthermore, Z-VAD-fmk was also able to confer protection against AG–4 induced apoptosis (Fig 4E) substantiating the role played by caspases.

As a measure of further revalidating and confirming the role of caspases in AG–4 induced apoptosis, we studied the specific cleavage of PARP (116 kDa), a well known substrate of caspase–3. Our results showed that in AG–4 treated (5.4 μM) U937 cells lysate, specific cleavage of PARP yielded 85 kDa cleaved fragment as obtained on analysis by western blotting with anti-PARP antibodies for different time duration (Fig 4F). A well noted function of PARP is its ability to help repair single-strand DNA nicks. Thus cleavage of PARP was found to be causing abatement of DNA repair process leading to further cellular damage.

### AG–4 caused oligonucleosomal degradation and cell cycle arrest

**Fig 5:**
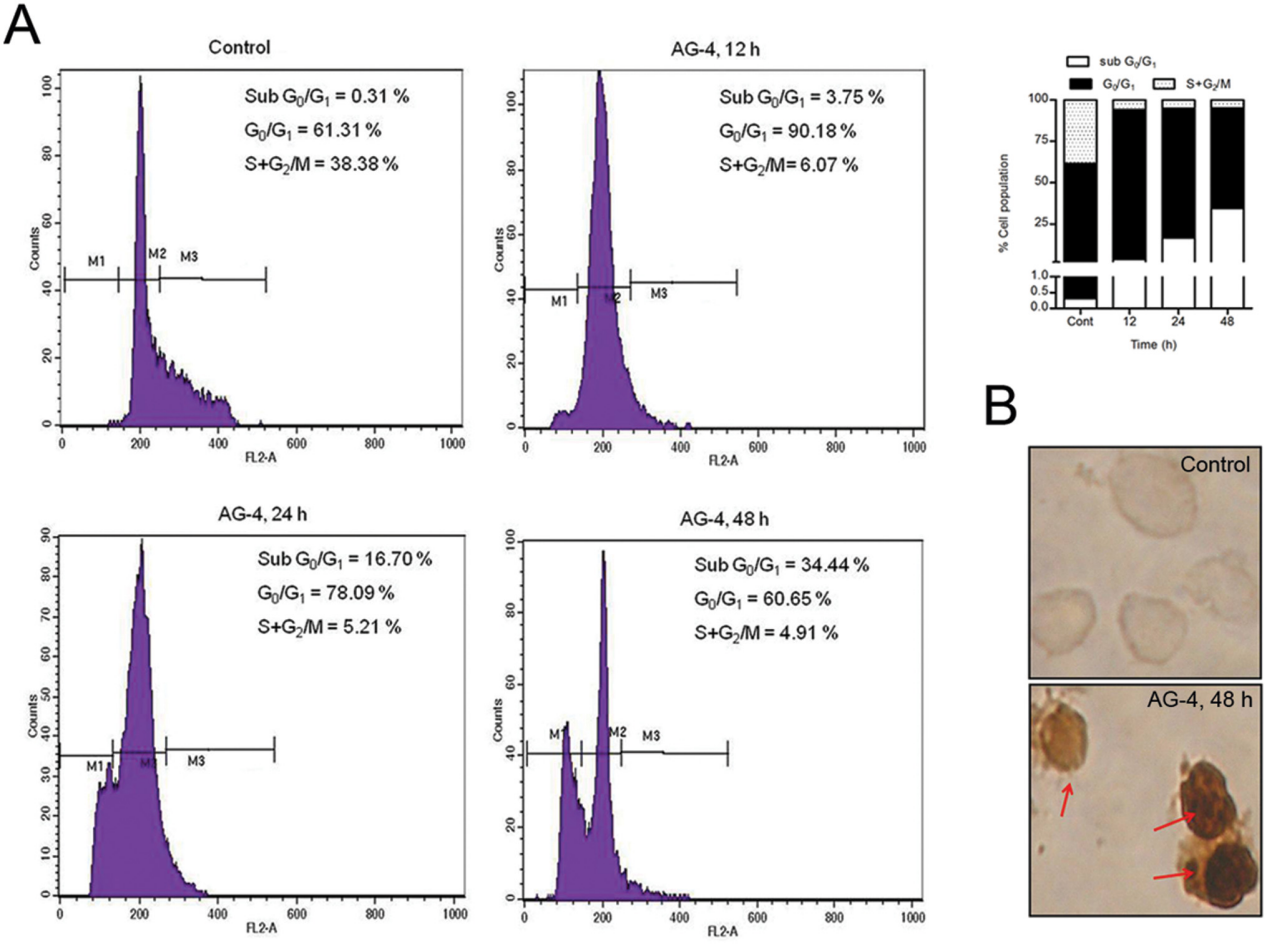
Effect of AG–4 on cell cycle progression and DNA degradation. (A) Flow cytometric analysis of cell cycle phase distribution of U937 cells treated with AG–4 (5.4 μM, 0–48 h). The percentage of sub G_0_/G_1_ cells was assessed using propidium iodide staining. Histograms depict percentage of cells in various phases of cell cycle. The figure is one representative of three independent experiments. (B) Analysis of TUNEL positivity. Control and AG–4 treated (5.4 μM, 48 h) U937 cells were stained as described in Materials and Methods. Cells were examined under light microscope (100X). Presence of DNA nicking is indicated by arrows. The figure is a representative profile of at least three experiments.

One of the terminal processes of apoptosis is DNA degradation largely mediated by caspases [34]. Since activation of caspases coupled with impairment of PARP was observed, we performed TUNEL assay to detect DNA fragmentation. In cells treated with AG–4 for 48 h, brown deposits indicating enshrined TdT-labelled nuclei were found (Fig 5B); representing nicking of DNA. The binding of FITC labelled dUTP to nicked ends via TdT was also quantified by flow cytometrically wherein the fluorescence obtained was directly proportional to the number of DNA nicks induced. U937 cells treated with AG–4 induced DNA fragmentation as evident from the binding of dUTP-FITC (data not shown).

One of the important therapeutic missions for development of new anticancer agents is to ascertain disturbance, if any of the cancer cell cycle. Perturbation of the cell cycle has proved to be a crucial target for development of new therapeutics [35]. To elucidate AG–4 induced mode of action, its effect on cell cycle progression was examined. The amount of dye that binds in a cell depends on its DNA content. The fluorescence intensity corresponds to fragmented DNA in apoptotic cells translates into a region lower than that of G_0_/G_1_ cells which is referred to as a sub G_0_/G_1_ peak. In AG–4 treated cells, a sharp rise in accumulation of apoptotic cells was observed in the sub G_0_/G_1_ region for increasing duration of treatment. Quantitative analysis of results obtained by us revealed that about 3.75%, 16.7% and 34.44% of the cells were obtained in the sub G_0_/G_1_ phase when treated with AG–4 for 12 h, 24 h and 48 h respectively (Fig 5A) implying significant cell cycle arrest.

### Altered expression of Atg proteins in AG–4 treated cells

**Fig 6:**
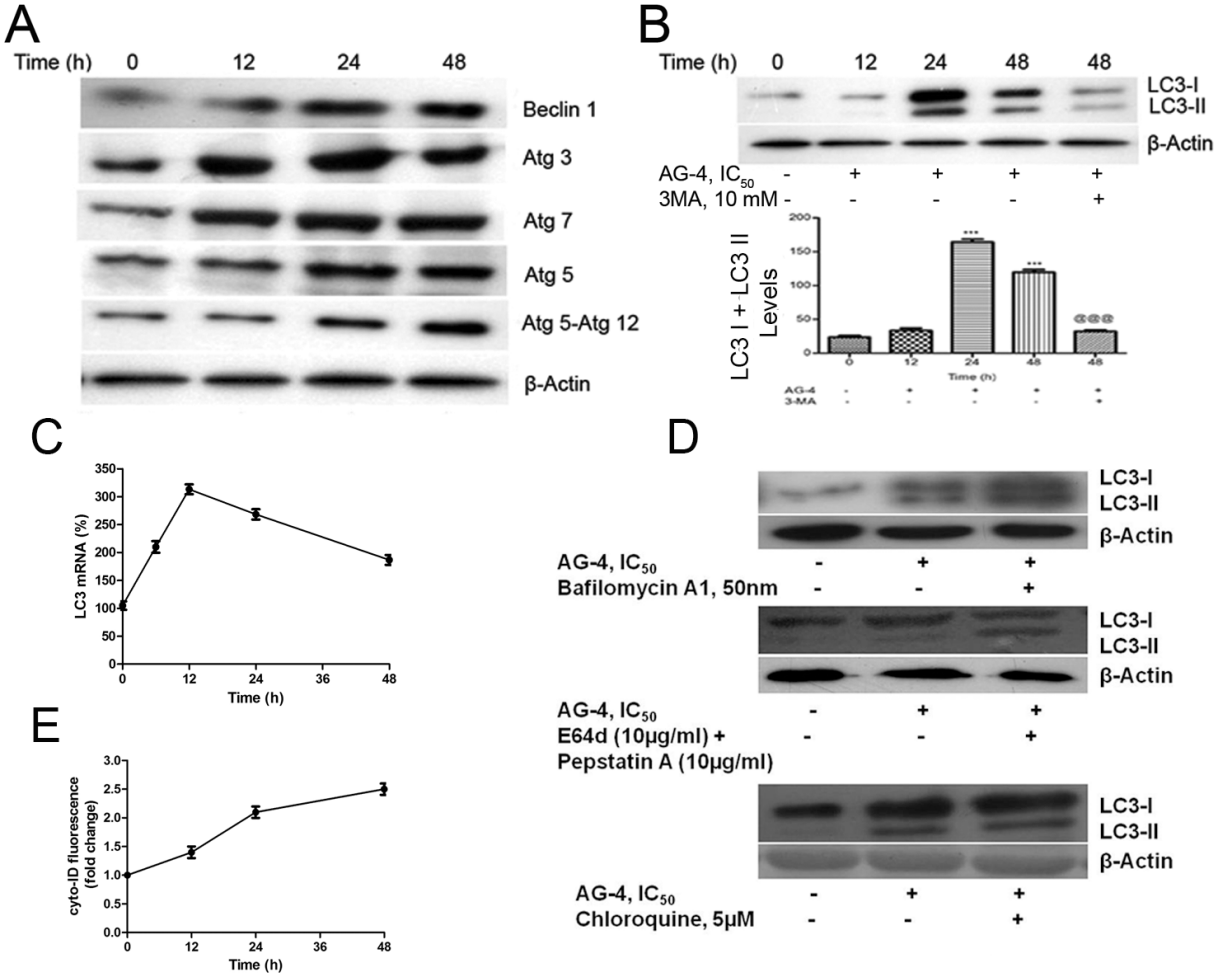
AG–4 alters expression levels of autophagic proteins and induces LC3 conversion. Whole cell extracts were made from control and AG–4 (5.4 μM, 0–48 h) treated cells and subjected to western blotting for (A) Atg proteins (B) LC3 processing. For the inhibition of autophagy, cells were pre-treated with known autophagy inhibitor, 3-MA (10 mM, 4 h) followed by treatment with AG–4 (5.4 μM, 48 h). (C) Expression of LC3 mRNA. Cells were treated with AG–4 and LC3 mRNA expressionwere examined by quantitative real-time PCR. (D) U937 cells were treated with AG–4 (5.4 μM, 0–48 h) [with or without co-treatment with Bafilomycin A1 (50 nM), E64d (10 μg/ml) plus pepstatin A (10 μg/ml) or Chloroquine (5 μM)]. Lysates were similarly prepared and subjected to western blotting. β-actin was used to ensure equal loading. The figure is a representative profile of three experiments. (E) Control and AG–4 treated U937 cells were stained with Cyto-ID and quantified by flow cytometry.

Besides its anti-proliferative functions, Bcl–2 might also modulate autophagy by forming an inhibitory complex with Beclin 1—which has a central role in autophagy [36]. Fig 6A demonstrated a distinct rise in expression of Beclin 1 with increased time of treatment. This was in accordance with the decline in Bcl–2 levels by AG–4. During the past years, molecular studies elucidated that the process of autophagy is critically mediated by a group of Atg proteins that function to formulate autophagosomes [37]. Accordingly, western blot expressions have brought to light increase in expressions of Atg 3, Atg 5, Atg 7 and the conjugated Atg 5-Atg 12 with increasing duration of AG–4 treatment (Fig 6A). These implied that AG–4 induced the occurrence of autophagy.

### AG–4 induced LC3 processing

Existence of microtubule-associated protein light chain 3 (LC3) in two forms, namely—cytosolic (LC3-I, 18 kda) and membrane bound (LC3-II, 16 kda) had been discussed in a number of recent investigations which also elaborated the correlation between the amount of LC3-II and the extent of autophagosome formation. Thus, the same is regarded as a hallmark of autophagy [38]. Immunoblotting analysis is done using anti-LC3 antibody demonstrated that levels of LC3-II and LC3-I protein increased significantly after 24 h and 48 h of treatment as compared to control (Fig 6B), Notably, pre-treatment with 3-MA (10 mM, 4 h), a known inhibitor of autophagy suppressed this increase in LC3-II expression. Furthermore, we checked the mRNA level of LC3, which is associated with the autophagosomes formation in response to autophagy. As shown in Fig 6C, LC3 mRNA expression was increased after treatment with AG–4 when compared with the controls. Earlier Literature suggests that LC3-II accumulation could be due to increased upstream autophagosome formation or impairment of downstream autophagosome-lysosome fusion [39]. To distinguish between these two possibilities, levels of LC3-II were checked in the presence of bafilomycin A1 (50 nM), E64D plus pepstatin A (10 μg/ml each) or chloroquine (5 μM), which inhibits downstream autophagosome-lysosome fusion and lysosomal proteases, respectively. AG–4 increased LC3-II levels in the presence of bafilomycin A1, E64d plus pepstatin A or chloroquine compared to only AG–4, bafilomycin A1, E64d plus pepstatin A or chloroquine (Fig 6D). To confirm autophagy induction after AG–4 treatment, U937 cells were stained with the Cyto-ID Green Detection Reagent. Cyto-ID serves as a selective marker of autolysosomes and early autophagic compartments. As shown in Fig 6E, AG–4 treatment induced an increase in Cyto-ID fluorescence after treatment in U937 cells. These results validate that AG–4 induced autophagic vacuolization is a consequence of autophagic flux activation.

### Formation of AVO in AG–4 treated U937 cells

**Fig 7:**
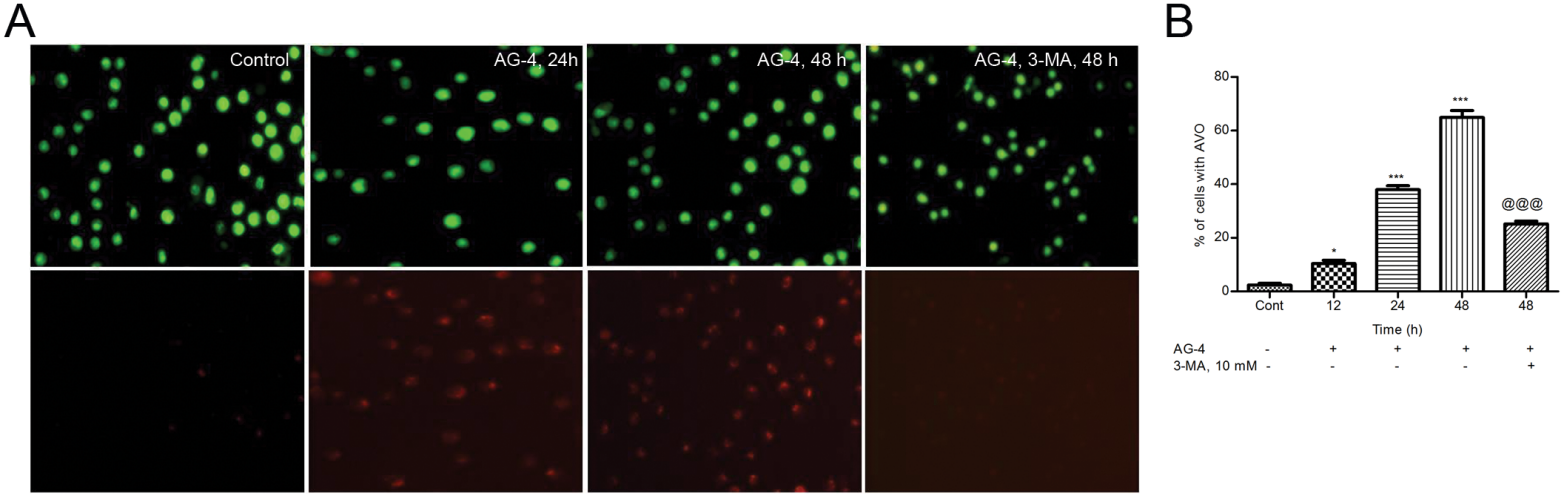
Detection of AVO in U937 cells. Control and AG–4 treated (5.4 μM, 0–48 h) U937 cells in the presence or absence of 3-MA (10 mM, 4 h) were stained with AO (1 μg/ml, 15 min) followed by flow cytometry for quantification or fluorescence microscopy. (A) Microphotograph of AVO. Detection of green and red fluorescence in AO stained cells was performed using a fluorescence microscope (60 X). At least 20 microscopic fields were observed for each sample. (B) Flow cytometric quantification of AVO. Histograms represent the percentage of cells with AVO and have been derived from at least three experiments (*p<0.05, ***p<0.001, compared to control cells; @@@ p<0.001, compared to only AG–4 treated cells).

To analyse the formation of AVO, which is a salient feature of autophagy, we used the lysosomotropic dye, AO that could traverse easily across biological membranes under uncharged conditions. However, its protonated form accumulates and forms aggregates in acidic compartments that emit bright red fluorescence [40]. As shown in Fig 7A, green fluorescence was primarily emitted in control cells with negligible red fluorescence, thereby highlighting a lack of AVO. On the other hand, U937 cells treated with AG–4 displayed a time dependent rise in red fluorescent AVO, maximum was found after 48 h of treatment. Cells stained by AO were further examined by flow cytometry to confirm and quantify the development of AVO. Fig 7B demonstrated that treatment with AG–4 (5.4 μM) increased the bright red fluorescence intensity in a time-dependent manner in U937 cells. 3-MA suppressed the induction of AVO in U937 cells treated with AG–4 (Fig 7A and 7B).

### AG–4 induced formation of autophagosomes

**Fig 8:**
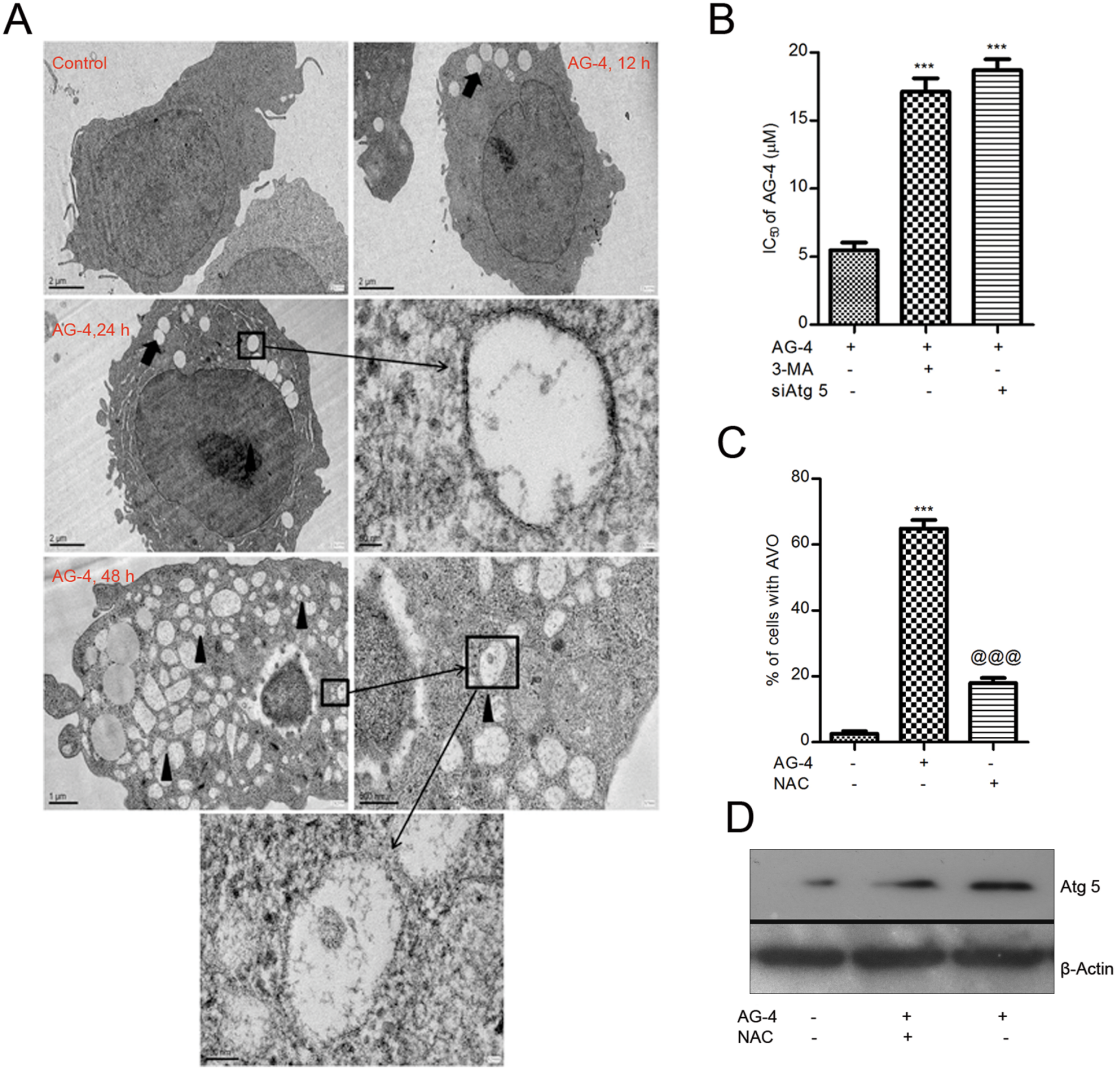
(A) TEM microphotographs of autophagosomes. Representative electron micrographs of control and AG–4 treated (5.4 μM, 0–48 h) U937 cells. Black arrows indicate autophagosomes and black arrow heads indicate autophagolysosomes including residual digested material. The figure is a representative profile of three experiments. (B) Contribution of autophagy in AG–4 induced cytotoxicity. Cells were pre-treated with 3-MA (10 mM, 4 h) or transfected with siAtg 5 for 72 h followed by treatment with AG–4 (0–50 μM, 48 h). Cell viability was determined by MTS-PMS assay. Results are expressed as IC_50_ (mean ± SEM) from three independent experiments (***p<0.001, compared to only AG–4 treated cells). (C, D) Effect of anti-oxidant on AG–4 induced autophagy. Cells were treated with AG–4 (5.4 μM, 48 h) in presence or absence of NAC (2.5 mM) followed by flow cytometry for quantification of AVO or immunoblotting for Atg 5 expression levels. (C) Histograms represent the percentage of cells with AVO and have been derived from at least three experiments (***p<0.001, compared to control cells; @@@ p<0.001, compared to only AG–4 treated cells). (D) The figure is a representative profile of at least three experiments.

Classically, the golden standard to highlight the formation of autophagosomes in cells is considered to be provided by transmission electron microscopy [41]. As shown in Fig 8A untreated U937 cells showed significantly uniform cytoplasm on our examination by electron microscopy. In contrast, after treatment with AG–4, cells showed ultra structures that were typical of autophagy. Under higher magnification, the double membraned autophagosomes were observed to coalesce with big and small single membraned vesicles. Some vacuoles also contained remnants of degraded organelles. After 48 h of treatment, almost the entire intracellular space was inundated by large vacuoles (Fig 8A). These observations indicated that AG–4 induced death in U937 cells with distinct autophagic morphology.

Autophagy has been paradoxically associated with both cell survival and cell death depending on the cellular context [42]. Hence, we next attempted to determine whether the autophagy induced by AG–4 served as a pro-survival or pro-death mechanism. Pretreatment of cells with 3-MA attenuated AG–4 induced cytotoxicity and its IC_50_ increased by 3 fold to 17.0 μM (Fig 8B). Atg 5 has been characterized as an ubiquitin-like protein involved in autophagosome formation [43]. Therefore, it led us to believe that the silencing of Atg 5 expression would result in a fall in AG–4 induced cytotoxicity. In U937 cells, the expression of Atg 5 was diminished after 72 h transfection of siRNA against Atg 5 (S2 Fig). The level of AG–4 induced cell death was also attenuated in Atg 5 silenced cells and its IC_50_ increased to 18.0 μM (Fig 8B). Altogether, this indicated that autophagy contributed to AG–4 induced death in U937 cells.

### AG–4 induced ROS triggered autophagy

ROS act as signalling molecules in growth and differentiation of cells [44,45]. ROS mediated autophagy has been observed in a number of different cancer cells [46]. Having already identified that AG–4 induced generation of ROS, we next assessed the role of ROS in AG–4 induced autophagy in U937 cells. To investigate a functional link between ROS production and autophagy induction, we co-incubated cells with antioxidant NAC (2.5 mM) and AG–4 (5.4 μM, 48 h) and examined its effect on AVO formation and Atg 5 expression. Attenuation of ROS levels by NAC significantly decreased the percentage of AVO (Fig 8C) compared to only AG–4 treated cells. Furthermore, there was a decline in Atg 5 expression upon AG–4 treatment in the presence of NAC as seen by immunoblotting methods (Fig 8D). Taken together, these data suggested ROS mediated induction of autophagy in U937 cells.

### AG–4 induced apoptosis and autophagy were dependent on each other

**Fig 9:**
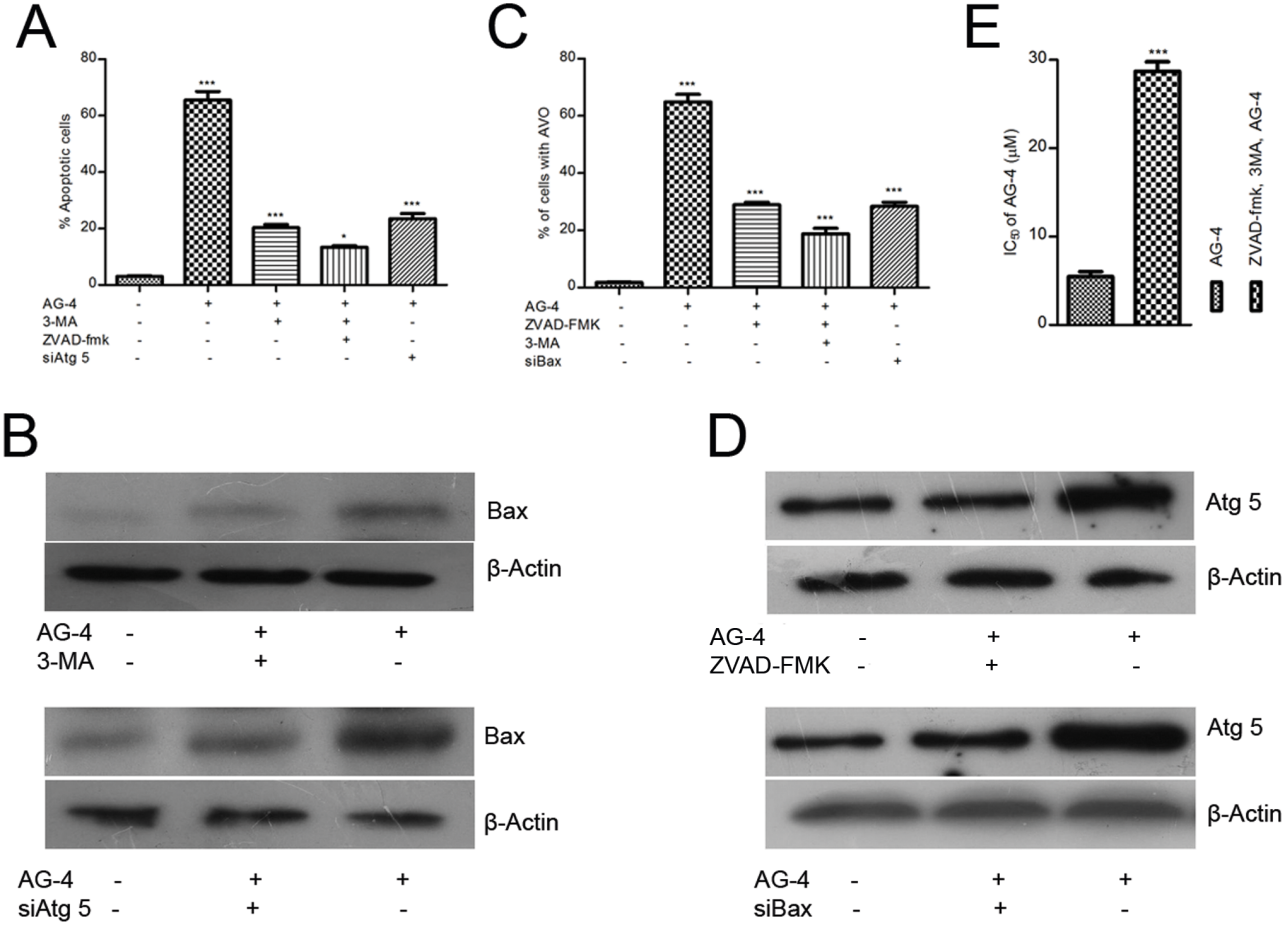
AG–4 induced apoptosis and autophagy are dependent on each other. (A, C) Effect of inhibitors on AG–4 induced Annexin V positivity and AVO formation. Cells were treated with Z-VAD-fmk (20 μM), 3-MA (10 mM, 4 h) or both Z-VAD-fmk and 3-MA or transfected with siAtg 5 or siBax followed by treatment with AG–4 (5.4 μM, 48 h). (A) Histograms depict percentage of apoptotic cells and are presented as the mean ± SEM from three independent experiments (***p<0.001, as compared with control; @@@p<0.001, as compared with only AG–4 treated cells). (C) Histograms depict percentage of cells with AVO and are presented as the mean ± SEM from three independent experiments (***p<0.001, as compared with control, @@@p<0.001, as compared with only AG–4 treated cells). (B, D) Effect of inhibitors on apoptotic and autophagic proteins. Cells were treated with Z-VAD-fmk (20 μM), 3-MA (10 mM, 4 h) or both Z-VAD-fmk and 3-MA or transfected with siBax or siAtg 5 followed by treatment with AG–4 (5.4 μM, 48 h). Whole cell lysates were prepared and subjected to immunoblot analysis using specific antibodies against Bax or Atg 5. Analysis was confirmed with three different sets of experiments. (E) Effect of simultaneous inhibition of apoptosis and autophagy on AG–4 induced cytotoxicity. Cells were treated with Z-VAD-fmk (20 μM) and 3-MA (10 mM, 4 h) followed by treatment with AG–4 (0–50 μM, 48 h). Cell viability was determined by MTS-PMS assay. Results are expressed as IC_50_ (mean ± SEM) from three independent experiments (***p<0.001, compared to only AG–4 treated cells).

The results described thus far show that AG–4 induced both apoptosis and autophagy in U937 cells. Recent studies have pointed towards a complex and circumstantial interplay between autophagy and apoptosis involved in the process of cell death induced by prospective anti-cancer moieties during which they may act independent of each other or may function as partners in a synchronized manner [47]. To determine the relationship between the two modes of PCD in the context of AG–4 mediated cytotoxicity, cells were treated with inhibitors of apoptosis and autophagy followed by treatment with AG–4 and subjected to Annexin V-PI assay, AVO quantification and western blot analysis. Annexin V-PI assay and western blot analysis were conducted on cells pre-incubated with 3-MA (10 mM, 4 h) followed by AG–4 (5.4 μM, 48 h) treatment. Fig 9A revealed a substantial downward trend in percentage of apoptotic cells in autophagy inhibited cells as against the same in only AG–4 treated cells. Furthermore, western blot analysis (Fig 9B) revealed that Bax (a crucial protein of the apoptotic pathway) levels decreased appreciably (compared to only AG–4 treated cells) after inhibition of autophagy by 3-MA. This was further corroborated by apoptosis assay and immunoblotting analysis of Atg 5 silenced cells which also depicted a significant decrease in percentage of apoptotic cells (Fig 9A) and Bax expression levels (compared to only AG–4 treated cells) (Fig 9B). Next, we co-incubated cells with Z-VAD-fmk (20 μM) and AG–4 (5.4 μM, 48 h) and subjected the same to flow cytometry for AVO quantification and western blot analysis to determine the levels of Atg 5, an essential protein involved in autophagy. The percentage of AVO was seen to have effectively decreased compared to only AG–4 treated cells (Fig 9C). Also, Atg 5 expression was repressed in Z-VAD-fmk treated cells compared to that in only AG–4 treated cells (Fig 9D). This was confirmed in Bax silenced cells which also exhibited decline in AVO formation (Fig 9C) and Atg 5 levels (Fig 9D). In addition, we determined the IC_50_ of AG–4 after simultaneously inhibiting apoptosis and autophagy using Z-VAD-fmk and 3-MA and then treated with AG–4 (5.4 μM; 48 h). Inhibitory concentration of AG–4 increased further to 30 μM (Fig 9E) and the proportion of apoptotic cells (Fig 9A) and AVO declined significantly compared to only AG–4 treated cells (Fig 9C). Thus, these results highlighted the fact that both apoptosis and autophagy contribute to AG–4 induced cytotoxicity and their effects depend on each other.

### AG–4 induced inhibition of the PI3K/Akt/mTOR pathway

The PI3K/Akt/mTOR pathway, an intracellular signalling network that is often constitutively hyperactivated in many types of cancer and is known to have prototypic functions in cellular proliferation, growth, differentiation and survival. Therefore, inhibition of the PI3K/Akt/mTOR signalling pathway can be looked into as a promising tool against cancer [48].

**Fig 10:**
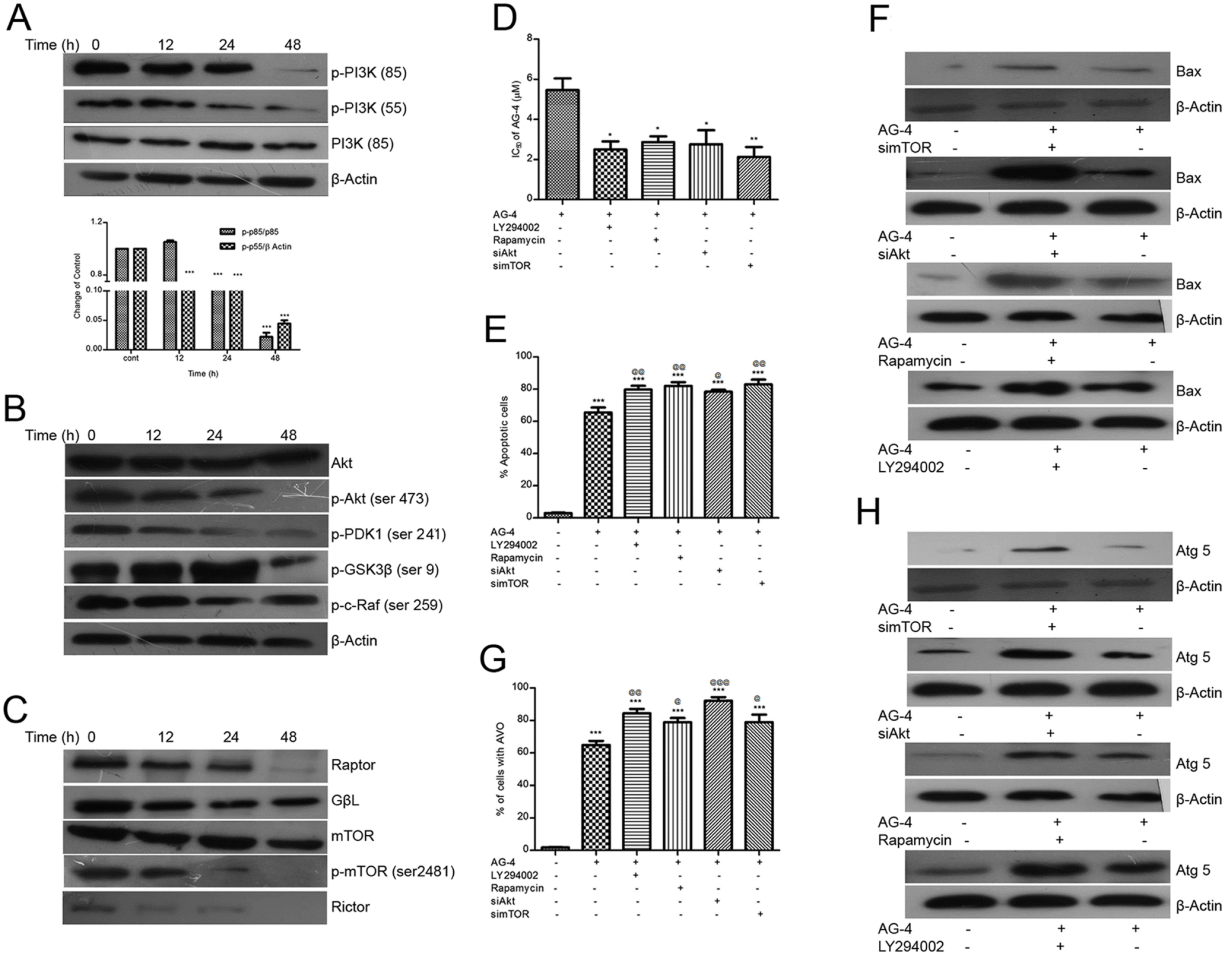
AG–4 inhibits PI3K/Akt/mTOR pathway. (A) Control and AG–4 treated (5.4 μM, 0–48 h) cells were analyzed by western blot for phosphorylated and total PI3K expression. The results shown are representative of three experiments. Histograms represent densitometric analysis of relative phosphorylation levels of PI3K. (B) Control and AG–4 treated (5.4 μM, 0–48 h) cells were analyzed by western blot for Akt pathway proteins. Analysis was confirmed with three different sets of extracts. (C) Control and AG–4 treated (5.4 μM, 0–48 h) cells were analyzed by western blot for mTOR pathway proteins. The figure is a representative profile of three experiments. (D, E, G) Effect of inhibitors on AG–4 induced cytotoxicity, Annexin V positivity and AVO formation. Cells were treated with LY294002 (20 μM, 1 h); Rapamycin (20 nm, 1 h) or transfected with siAkt, simTOR followed by treatment with AG–4 (D) Cell viability was assessed by MTS-PMS assay. Data are presented as IC_50_ (mean ± SEM) from three independent experiments (*p<0.05, **p<0.01, as compared with only AG–4 treated cells). (E) Histograms depict percentage of apoptotic cells and are presented as the mean ± SEM from three independent experiments (***p<0.001, as compared with control; @@p<0.01 & @p<0.05, as compared with only AG–4 treated cells). (G) They were then analysed for AVO by AO staining. Histograms depict percentage of cells with AVO and are presented as the mean ± SEM from three independent experiments (***p<0.001, as compared with control; @@@p<0.001, @@p<0.01 & @p<0.05, as compared with only AG–4 treated cells). (F, H) Effect of inhibitors on apoptotic and autophagic proteins. Cells were treated with LY294002 (20 μM, 1 h), Rapamycin (20 nm, 1 h) or transfected with siAkt, simTOR followed by treatment with AG–4 (5.4 μM, 0–48 h). Whole cell lysates were prepared and subjected to immunoblot analysis using specific antibodies against Bax or Atg 5. Analysis was confirmed with three different sets of experiments.

The expression of phosphorylated-PI3K (p-PI3K) was found to have significantly decreased in AG–4 treated cells both in phosphorylated forms of 85 and 55 PI3K when compared to control, in a time dependent manner (Fig 10A). Densitometric analysis revealed that the ratios of p-PI3K (p85)/total PI3K evidenced a time-dependent decrease after AG–4 treatment by 78.9–97.8% as against the control cells (***p<0.001) (Fig 10A).

On further examination of the inhibitory effect, if any, of AG–4 on Akt, phosphorylation of Akt, its downstream substrate—GSK 3β and upstream activator–PDK revealed that AG–4 inhibited phosphorylation of Akt, GSK 3β and PDK1 at Ser473, Ser9 and Ser241 respectively as shown in Fig 10B. The levels of total Akt remained unaltered. Akt phosphorylates inhibitory site (Ser259) of c-Raf, a crucial molecule of MAPK/ERK signal transduction pathway [49]. In accordance with decreased phosphorylation of Akt, AG–4 was able to inhibit the phosphorylated levels of c-raf (Fig 10B). These observations prompted us to infer that downregulation of PI3K/Akt might have a crucial role in AG–4 induced cytotoxicity. In order to ascertain and confirm such a possibility, cells were co-incubated with AG–4 and the PI3K inhibitor LY294002, and cell viability was determined. Cell viability decreased from 5.4 μM to 2.5 μM (Fig 10D). This was confirmed in cells in which Akt had been silenced following 48 h transfection using siRNA targeting Akt (S2 Fig). The level of AG–4 induced cell death was also enhanced in Akt silenced cells as evident from its lowered IC_50_ (Fig 10D).

Since phosphorylation of Akt at Ser473 is a positive regulator of mTOR signaling pathway [50], we examined the effect of AG–4 treatment on mTOR activity. Treatment of U937 cells with AG–4 led to decrease in the levels of the phosphorylated (activated) form of mTOR (Ser2481), while total mTOR level was not affected by the treatment (Fig 10C). The data also indicated that the level of Raptor and Rictor, the companion proteins of mTORC1 and mTORC2, respectively, was also downregulated by AG–4. Furthermore, the level of G protein β-like protein (GβL), which constitutes a part of both mTORCs, was downregulated by AG–4 treatment (Fig 10C). We endeavoured to further analyse the effects of mTOR inhibition on the anti-proliferative potential of AG–4, cells were pre-treated with rapamycin (20 nm, 1 h) followed by exposure to AG–4. Relevant MTS-PMS assay showed enhancement of the cytotoxic potential of AG–4 on inhibition of mTOR, IC_50_ was seen to have decreased to 2.8 μM (Fig 10D). This was substantiated by silencing mTOR in cells by 48 h transfection using siRNA. AG–4 induced cytotoxicity was augmented in mTOR silenced cells (Fig 10D). Taken together, these results clearly expressed the contribution of PI3K/Akt/mTOR inhibition in AG–4 mediated course of action.

To further determine the functional significance of PI3K/Akt/mTOR pathway suppression by AG–4, we assessed the role of Akt or mTOR in AG–4 mediated apoptosis and autophagy. Percentage of apoptotic cells and Bax level showed a sharp rise in cells treated with LY294002 or Akt silenced cells implying increased apoptosis in Akt inhibited cells (Fig 10E and 10F). Also, percentage of AVO and Atg 5 expression was enhanced in Akt inhibited cells (Fig 10G and 10H)–signifying increased autophagy. Similarly, increased percentage of apoptotic cells and Bax level were observed in cells treated with Rapamycin or mTOR silenced cells (Fig 10E and 10F). Percentage of AVO and Atg 5 expression levels increased after inhibition of mTOR (Fig 10G and 10H). Thus, our findings unequivocally substantiated the fact that suppressing PI3K/Akt/mTOR pathway was indispensable for AG–4 mediated apoptosis and autophagy.

## Discussion

Our study disseminated novel proof demonstrating induction of multiple modes of cell death and their interconnection in the context of Andrographolide analogue induced cytotoxicity in U937 human leukemic cells.

Prevalent therapies for cancer generally comprise: (a) surgical treatment by way of removal of solid tumor masses; (b) chemical (chemotherapy) treatment using chemotherapeutic agents; or (c) physical (radiotherapeutic) treatment. However, several drawbacks noticed over the years in case of chemotherapy based on agents inducing only apoptosis has rendered its efficacy quite limited as treatment modalities. Recent studies have revealed that induction of apoptosis no longer the sole tenet of cancer treatment. On the contrary, non apoptotic mechanisms (such as autophagy) are increasingly being implicated to achieve cell death by chemotherapy. Thus, a strategy involving a combined and coherent action of apoptotic as well as non- apoptotic programmes might lead to an effective treatment mechanism resulting in a minimisation of the chance of relapse [51].

We sought to identify novel agents by examining natural products as traditional medicinal plants bear a long history of safe treatment and their bioactive molecules had been generally found to be non-toxic or with low and negligible toxicity to human [52]. We focussed our earlier study on the Andrographolide analogue AG–4, previously abbreviated as **6a**, which demonstrated lower inhibitory concentration than parent Andrographolide and induced apoptosis in U937 human leukemic cells [20]. Nevertheless, we had since thought it proper to explore further the molecular process underlying AG–4 induced cell death. Therefore, we endeavoured to explore the molecular mechanisms underlying AG–4 induced cell death in U937 cells, and examined the intricate relationship between AG–4 induced apoptosis and autophagy.

In our previous publication [20], we had shown that AG–4 was most effective in U937 cells. In our present experiment, we extended the scope of our work and investigated its anti-proliferative activity in some more cell lines (Raji, HCT–15, MCF–7) and found that AG–4 exhibited the least IC_50_ in U937 cells (Fig 1A and 1B). Even at a concentration of 50 μM, AG–4 did not affect healthy human PBMC (Fig 1B) which substantiated the fact that AG–4 did not produce any deleterious impact on healthy cells. This was in conformity with the previously published data thereby showing that the inhibitory concentration of AG–4 in NIH3T3 and L132 cells were substantially higher than that of 5.4 μM (IC_50_ in U937 cells). Induction of apoptosis was a highly desirable characteristic for screening of chemotherapeutic drugs. AG–4 proved to be a potent inducer of apoptosis in U937 cells as evidenced by Annexin V positivity and nuclear changes [20]. Apoptosis, a morphologically distinct form of PCD was well orchestrated by a set of hierarchical molecular events and followed an evolutionarily conserved mechanism. We next studied the events governing the apoptotic process.

It is known that cancer cells generate higher levels of endogenous ROS stress due to genetic instability, elevated metabolic rates, and mitochondrial dysfunction. Since ROS beyond a threshold level could inflict cellular damage, it could be hypothesized that compounds effecting enhancement of ROS generation as we believe that it could facilitate apoptosis in cancer cells by elevating free radical induced DNA damage [53]. Our results showed that AG–4 was able to trigger the level of ROS in a time-dependent manner (Fig 2A) accompanied by diminishing cellular thiol levels (Fig 2D), resulting in changes in the redox potential. However, ROS generation was not significant in AG–4 treated PBMC further substantiating its non-toxicity towards healthy cells. Furthermore, the generation of ROS in response to AG–4 was supported by the finding that co-treatment with NAC, a general antioxidant, blocked the oxidation of CM-H_2_DCFDA. The significant role of ROS in AG–4 mediated apoptosis was justified by the abatement of its anti-proliferative and apoptotic effects on application of NAC (Fig 2B and 2C). Such findings further corroborated the fact that susceptibility to anti-cancer drugs at the cellular level *in vitro* was determined by the cellular level of ROS and it might play a significant role in predicting chemotherapeutic efficacy and prognosis caused the cells to be susceptible to therapeutic agents and thus determined its efficacy.

In earlier studies, it was mainly emphasized that stimuli such as anti-cancer compounds were responsible for producing ROS leading to mitochondria initiated apoptosis by inducing loss of mitochondrial membrane potential [53]. Our results revealed that AG–4 treatment of U937 cells caused a decline in MMP and an increase in cytosolic calcium level (Fig 3A and 3B). Uncoupling of mitochondrial oxidative phosphorylation is caused by excessive free cytosolic Ca^2+^ which is instrumental in directing the cells to follow the executionary part of apoptosis. A wide range of anti-cancer drugs were known for their inhibitory action on growth of cancer cell by modulating the expression of apoptosis regulatory protein. As per available research reports, pro-apoptotic (Bax and Bad) and anti-apoptotic (Bcl–2 and Bcl-xl), members of Bcl–2 family, could affect mitochondrial membrane permeability [54]. Bax, usually found in cytosol, assisted in the release of cytochrome c from mitochondrial intermembrane space into cytosol by associating itself with outer membrane which induced its permeability. Bax function got inhibited by Bcl-xl [55]. Bcl–2 acted towards preserving mitochondrial integrity, suppressing release of cytochrome c and subsequently inhibiting apoptosis. The said pro- and anti-apoptotic proteins were considered to be exerting their control over apoptosis by way of forming necessary heterodimers among them resulting in mutual neutralization of bound pro and anti- apoptotic protein [56]. Furthermore, published reports revealed also that apoptosis induced by ROS was suppressed by Bcl–2 while enhancement in ROS generation was the result of over expression of pro-apoptotic Bax [57]. Therefore, striking a balance between the levels of expression of pro- and anti-apoptotic proteins was of paramount importance for causing survival or death of cells. We observed in the course of our in-depth study that AG–4 induced apoptosis was accompanied by disruption of MMP and release of cytochrome c (Fig 3F) through appropriate up-regulation of Bax, Bad and down regulation of Bcl–2 and Bcl-xl (Fig 3C). The fact that cytotoxic and apoptotic abilities of AG–4 were greatly diminished in Bax silenced cells signified the crucial contribution of Bax in AG–4 induced apoptosis (Fig 3D and 3E). Furthermore, the most prominent anti-apoptotic members of Bcl–2 family (e.g. Bcl–2, Bcl-xl) were duly identified and found to be overexpressed in leukemic cells [58]. Therefore; it was of great significance to find out and appreciate the dexterity and tremendous ability of AG–4 to induce apoptosis in U937 cells by way of down regulating Bcl–2 and Bcl-xl expression levels.

By virtue of loss of MMP and release of cytochrome c, cells reached a “point of no return” but yielded apoptosis through activation of caspase cascade [32]. Caspases, a group of cysteine-aspartic proteases were involved in the executionary part of apoptosis [59]. The activation of initiator caspases (e.g. caspase–8, -9) by apoptotic stimulation cleaved and thereby, activated executioner caspases (e.g. caspase–3), resulting in small and large sub-units arising out of such cleaving. A caspase cascade occurred leading to PARP (DNA repair enzyme and a known caspase substrate) cleavage and DNA degradation. In our study, activation of caspase–8, -9 and -3 was observed in AG–4 treated cells (Fig 4A–4C). If caspase activation contributed towards AG–4 induced cell death, caspase inhibition should protect against the drug induced death. Consistent with the idea, co-incubation with the pan-caspase inhibitor Z-VAD-fmk had been found to confer protection against AG–4 induced cytotoxicity and apoptosis (Fig 4D and 4E). Furthermore, the presence of cleaved PARP following AG–4 treatment (Fig 4F) corroborated caspase activation. Researchers showed that apoptosis eventually led to DNA fragmentation which was predominantly mediated by caspases. In accordance with the said finding, results of our TUNEL assay revealed evidences of fragmented DNA in AG–4 treated cells (Fig 5B). Furthermore, cell cycle arrest could be a crucial cellular response to DNA fragmentation before cells moved towards repair or death [35]. Since DNA repair enzyme PARP was inactivated, the cells were unable to repair the fragmented DNA eventually leading to arrest of cell cycle progression at sub G_0_/G_1_ phase (Fig 5A).

For almost two decades, Bcl–2, Bcl-xl (anti apoptotic protein) had been perceived to be acting by way of inhibiting one specific form of PCD—apoptosis. Nearly all of the impacts of Bcl–2, Bcl-xl on cancer were attributable to their effects on the apoptotic pathway. Apart from inhibiting apoptosis by binding to and interfering with the action of the pro-apoptotic proteins (Bad, Bax), Bcl–2, Bcl-xl also indirectly modulated autophagy [3]. Autophagy, denoted a catabolic process, caused lysosomal degradation of cytoplasmic contents in eukaryotes. The process of autophagy could be divided into four principal steps: (a) initiation involving the formation of phagosphore; (b) nucleation; (c) elongation, i.e., phagosphore expansion enclosing the material to be degraded, forming a double-membrane autophagosome; (d) maturation and degradation involving fusion of autophagosomes with lysosomes to form autolysosomes in which the luminal content is degraded by lysosomal acidic hydrolases [19]. Beclin 1, first identified as a Bcl–2 interacting protein, formed a part of a Class III PI3K complex that helped in the nucleation step of autophagosome formation, and recruited other Atg proteins to the pre autophagosomal membrane for its elongation. Beclin 1, also considered as a haploinsufficient tumor-suppressor gene, that was often monoallelically deleted in many types of cancer, signifying its vital role in autophagic cell death during cancer therapy [60]. As per our present study, AG–4 was found to be able to increase Beclin 1 levels with increased treatment times (Fig 6A) and this was consistent with suppressed levels of Bcl–2. Two ubiquitin-like conjugation systems were necessary for the elongation of autophagic vesicle. One pathway involved the conjugation of Atg 5 and Atg 12 arising out of usage of E1-like enzyme Atg7 [47]. Following treatment with AG–4, there had been considerable increase in levels of Atg 7, Atg 5 and the conjugated form of Atg 5-Atg 12 (Fig 6A). The other pathway involved induction of cytosolic LC3-I and its conjugation with phosphatidylethanolamine resulting in its conversion to autophagosome-associated LC3-II form in which Atg 3 was found to be having a crucial role to play. Hence, the processing of LC3-I to LC3-II was considered a hallmark of autophagy [47]. Treatment with AG–4 had been found to have increased Atg 3 levels inducing the processing of LC3 (Fig 6A and 6B). This was substantiated the fact that increased levels of mRNA of LC3 after AG–4 treatment (Fig 6C). Furthermore, it was observed that upon inhibition of lysosomal degradation by inhibitors (bafilomycin A1, E64D plus pepstatin A or chloroquine), the accumulation of LC3-II was not affected in AG–4 treated cells. These findings corroborated that the increase in LC3 level by AG–4 resulted in autophagy induction and not due to lysosomal degradation (Fig 6D). Cells engaged in autophagy did provide us with another vital characteristic feature which was the formation of AVO (or autophagolysosome) following the fusion of autophagosome with lysosomes [27]. The marked increase in the presence of AVO following AG–4 treatment was observed using AO staining which emitted red fluorescence under acidic conditions (Fig 7A and 7B). Ultrastructures showing autophagosomes and autophagolysosomes in AG–4 treated cells further substantiated our findings (Fig 8A). Therefore, all these observations did provide a strong evidence for a complete autophagy process upon AG–4 treatment. It would not be out of place to mention here that our study had perhaps been the first of its kind to show simultaneous induction of multiple modes of cell death by Andrographolide analogue. The role played by autophagy in cancer treatment was circumstantial having regard to an important understanding that autophagy acted as a double-edged sword in cancer. To tide over adverse conditions induced by stress from anti-cancer therapies, cancer cells might undergo autophagy that sequestered cellular components into autophagic vesicles as part of the survival response to stress [18]. On the contrary, autophagy played a crucial part in annihilating malignant cells by triggering an alternative pathway of cell death [61]. Whether autophagy triggered by AG–4 in U937 cells represented a mechanism for survival or cell death constituted a vital aspect of our study by applying 3-MA, a chemical inhibitor of autophagy. Our results indicated that 3-MA could reverse the cytotoxic effect of AG–4 (Fig 8B). This finding was further validated by silencing of Atg 5, a key molecule involved in autophagosome formation (Fig 8B), suggesting that AG–4 induced autophagy contributed to its cytotoxicity. Besides its role in apoptosis, there was accumulating evidence for autophagic processes in response to ROS. Co-treatment with NAC decreased percentage of AVO and repressed expression of Atg 5 (Fig 8C and 8D) signifying ROS mediated autophagy after AG–4 treatment.

Furthermore, the impact of apoptotic and autophagic responses to cell death and their interconnection differed with cell types and depends on the cellular environment [51]. Autophagy could act synergistically or as an antagonist to apoptosis in the process of cell death. If they acted as partners for inducing cell death, they might do so independently or might co-operate with one another. Thus, the interplay between these two modes of cell death was observed by us to be quite complex and circumstantial, but crucial to the fate of cells [7]. Therefore, the induction of both autophagy and apoptosis by AG–4 prompted us to investigate the interconnection between these two cellular processes. Results of our study revealed that down regulation of autophagy (by pharmacological or genetic inhibition) resulted in decreasing apoptosis (Fig 9A and 9B). On the other hand, inhibition of apoptosis (by pharmacological or genetic inhibition) also resulted in suppression of autophagy (Fig 9C and 9D). In addition, simultaneous inhibition of apoptosis and autophagy further reduced the cytotoxic, apoptotic and autophagic effects of AG–4 (Fig 9A–9E). Thus, apoptosis and autophagy both contributed and facilitated each other in the context of AG–4 induced cytotoxicity.

For the past few years, considerable progress had been made towards the elucidation of many cancer-related signalling pathways. Efforts had been underway to exploit this knowledge so as to target specific signal transduction molecules and achieve effective modes of treatment. Among the survival signals, PI3K/Akt/mTOR pathway had been reported to be playing a critical role in the pathogenesis progression of leukemia. A deregulation of this signalling pathway fostered the survival and proliferation of hematopoietic progenitor cells [48]. The present results as per our study indicated that AG–4 regulated p-PI3K, p-Akt, p-mTOR, as well as other key molecules p-PDK1, p-c-Raf and p- GSK 3β of PI3K/Akt/mTOR pathway (Fig 10A–10C). Akt specific inhibitor LY294002 or Akt gene silencing by siRNA caused further decrease in cell viability (Fig 10D). Similarly, mTOR inhibitor Rapamycin or siRNA against mTOR was seen to have increased the potency of AG–4 (Fig 10D) illustrating the inhibitory role of PI3K/Akt/mTOR pathway in AG–4 induced cytotoxicity. Apoptosis and autophagy were regulated by many common factors and both pathways shared several factors that were critical for their respective function—PI3K/Akt/mTOR signaling pathway reported to be one of them [62]. The activation of this pathway allowed cells to inhibit apoptosis and autophagy, which might be responsible for malignant transformation and increased rate of proliferation. Furthermore, PI3K activated its downstream target AKT which protected cells from apoptosis and thereby contributed to cellular survival. On the other hand, activated Akt also stimulates mTOR leading to inhibition of autophagy [63]. Besides emerging as a key negative regulator of autophagy, mTOR had also proved to be a potential regulator of apoptosis in various cancer cells [64]. Consistent with these facts, our results revealed that LY294002 or AktsiRNA significantly increased the apoptosis and autophagy induced by AG–4 (Fig 10E–10H). In addition, Rapamycin or mTORsiRNA significantly enhanced AG–4 induced apoptosis and autophagy (Fig 10E–10H). Therefore, this implied that AG–4 mediated autophagy and apoptosis correlated with suppression of PI3K/Akt/mTOR pathway.

Taken together, these findings provided the evidence that Andrographolide analogue AG–4 was most potent in human leukemic U937 cells with low toxicity to normal healthy cells. Further studies demonstrated that AG–4 induced cytotoxicity involved redox imbalance and apoptosis by inducing mitochondrial depolarisation and activation of the caspase cascade associated with blocking of PI3K/Akt/mTOR pathway. Moreover, AG–4 was found to be inducing autophagic cell death by promoting autophagosome formation, LC3 conversion and inhibition of PI3K/Akt/mTOR pathway, suggesting the role of both autophagy and apoptosis in AG–4 induced action. There had been reports demonstrating autophagic effects of Andrographolide in cancer cells [18,19]. Zhou et al. [19] had shown that Andrographolide sensitized cisplatin induced apoptosis via suppression of autophagy. However, the relationship between apoptosis and autophagy induced cell death in the context of Andrographolide induced cytotoxicity was yet to be looked into. Thus, this study of ours was perhaps the first to critically analyse and determine the various modes of cell death induced by Andrographolide analogue and their inter-relationship. The mechanism underlying AG–4 induced apoptotic and autophagic programmes having been deciphered in our study, further studies into its pre-clinical and clinical aspects may be undertaken to harness this promising molecule as a potential chemotherapeutic agent targeting PCD.

## Supporting Information

S1 Fig Scheme for chemical synthesis of AG–4. (PDF)

S2 Fig U937 cells were transfected with Bax siRNA (48 h) or Atg–5 siRNA (72 h) or Akt siRNA (48 h) or mTOR siRNA (48 h) and the expression levels were analysed by western blot analysis. The results shown are representative of three experiments.
(PDF)

## Abbreviations

3-MA: 3-methyladenine
AO: acridine orange
Atg: autophagy related
AVO: acidic vesicular organelles
CMFDA: 5-Chloromethylfluorescein Diacetate
CM-H_2_DCFDA: 5-(and–6)-chloromethyl–2',7'-dichlorodihydrofluorescein diacetate
DCF: 2',7'-dichlorofluorescein; EGTA, ethylene glycol tetraacetic acid
FACS: fluorescence activated cell sorter
FBS: heat inactivated fetal bovine serum
FITC: fluorescein isothiocyanate
GMFC: geometric mean fluorescence channel
JC–1: 5,5’,6,6’-tetrachloro–1,1’,3,3’-tetraethylbenzimidazolylcarbocyanine iodide
LC3: microtubule-associated protein light chain 3
MMP: mitochondrial transmembrane potential
mTOR: mammalian target of rapamycin
MTS: 3-(4,5-dimethylthiazol-2-yl)-5-(3-carboxymethoxyphenyl)-2-(4-sulfophenyl)-2H-tetrazolium, inner salt
NAC: N-Acetylcysteine
PARP: poly(ADP-ribose) polymerase
PBMC: human peripheral blood mononuclear cells
PBS: phosphate buffered saline
PCD: Programmed cell death
PI3K: phosphatidylinositol 3-kinase
PMS: phenazinemethosulphate
ROS: reactive oxygen species
siRNA: small interfering RNA
TEM: transmission electron microscopy
TUNEL: Terminal DeoxyribonucleotidylTransferase-Mediated dUTP Nick-End Labeling
VDAC1: Voltage-dependent anion –selective channel proteins 1
Z-VAD-fmk: Z-Val-Ala-DL-Asp (methoxy)-fluoromethylketone
