# Supplementary figures and images for: Andrographolide Analogue Induces Apoptosis and Autophagy Mediated Cell Death in U937 Cells by Inhibition of PI3K/Akt/mTOR Pathway
Source: PLoS One. 2015 Oct 5;10(10):e0139657. doi: 10.1371/journal.pone.0139657 (PMC4593644; doi:10.1371/journal.pone.0139657)

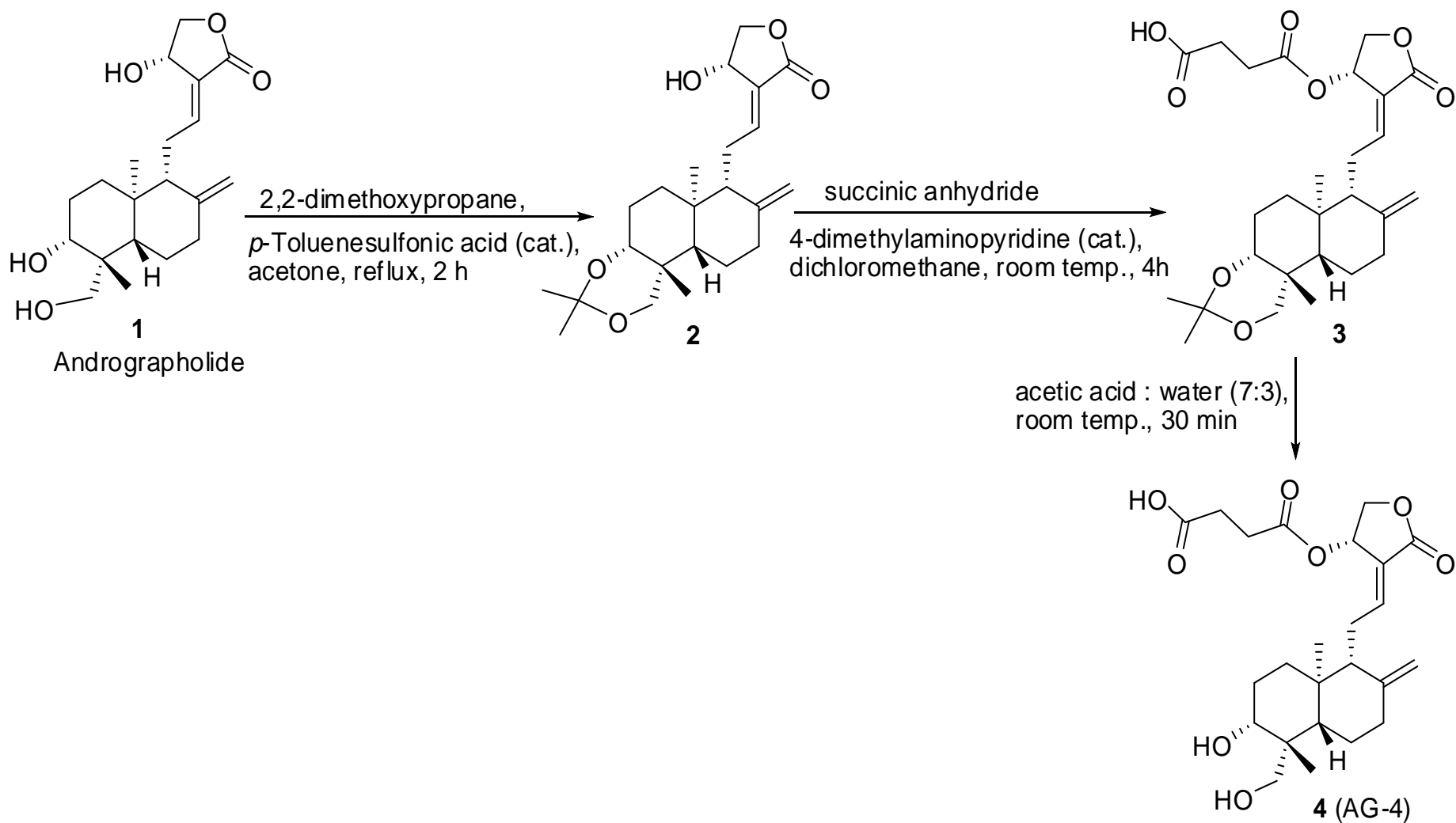

**Supplementary Figure 1.** Scheme for chemical synthesis of AG-4

Supplement: S1 Fig — (PDF) [file pone.0139657.s001.pdf]
