# Supplementary material for: Andrographolide Analogue Induces Apoptosis and Autophagy Mediated Cell Death in U937 Cells by Inhibition of PI3K/Akt/mTOR Pathway
Source: PLoS One. 2015 Oct 5;10(10):e0139657. doi: 10.1371/journal.pone.0139657 (PMC4593644; doi:10.1371/journal.pone.0139657)
Supplement: S2 Fig — The results shown are representative of three experiments. (PDF) [file pone.0139657.s002.pdf]

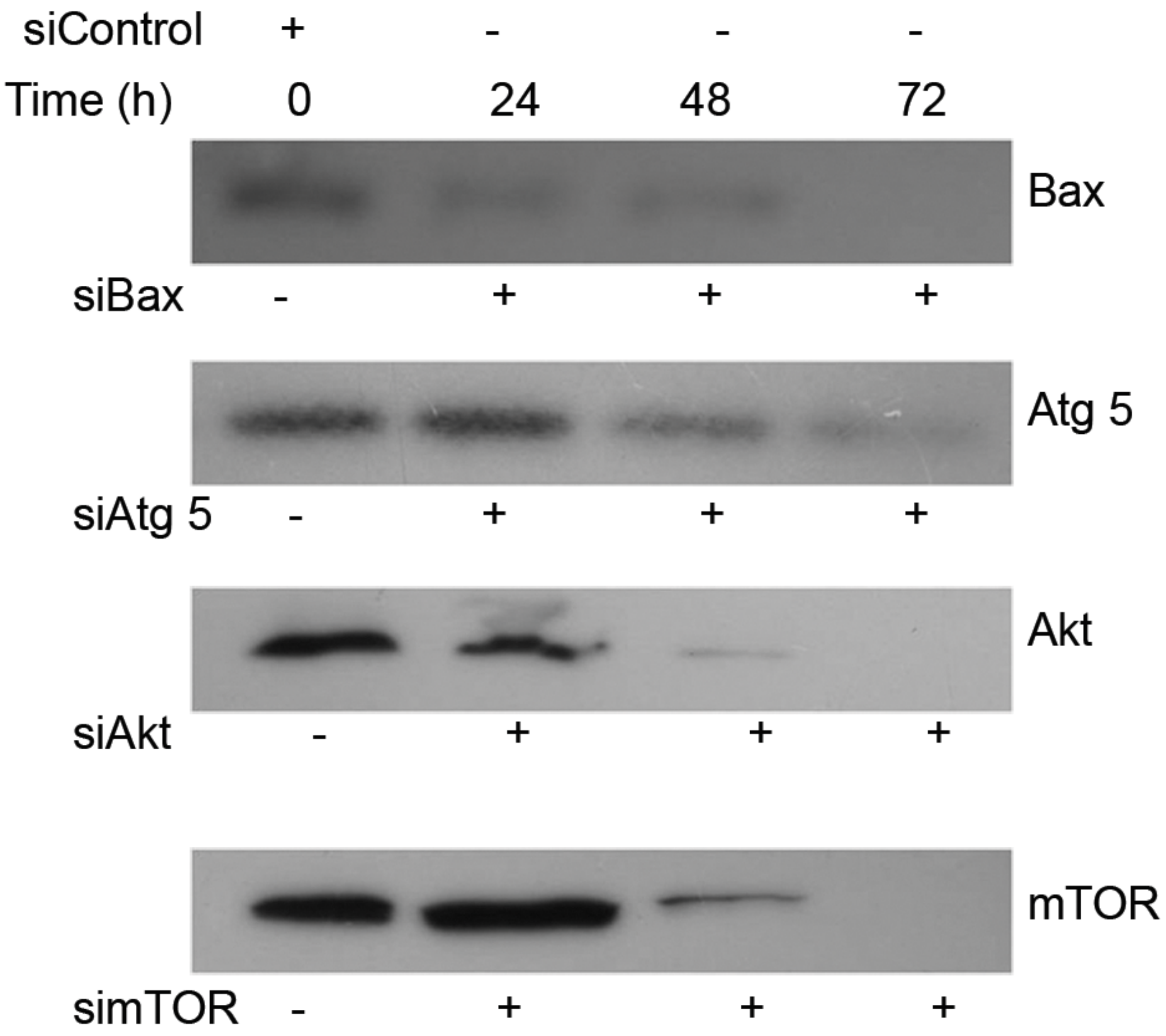

Supplementary Figure 2. U937 cells were transfected with Bax siRNA (48 h) or Atg-5 siRNA (72 h) or Akt siRNA (48 h) or mTOR siRNA (48 h) and the expression levels were analysed by western blot analysis. The results shown are representative of three experiments.
